# Evidence of covalent synergy in silicon–sulfur–graphene yielding highly efficient and long-life lithium-ion batteries

**DOI:** 10.1038/ncomms9597

**Published:** 2015-10-26

**Authors:** Fathy M. Hassan, Rasim Batmaz, Jingde Li, Xiaolei Wang, Xingcheng Xiao, Aiping Yu, Zhongwei Chen

**Affiliations:** 1 Department of Chemical Engineering, University of Waterloo, Waterloo, Ontario, Canada N2L3G1; 2 Chemical and Materials Systems, General Motors Global Research and Development Center, Warren, Michigan 48090, USA

## Abstract

Silicon has the potential to revolutionize the energy storage capacities of lithium-ion batteries to meet the ever increasing power demands of next generation technologies. To avoid the operational stability problems of silicon-based anodes, we propose synergistic physicochemical alteration of electrode structures during their design. This capitalizes on covalent interaction of Si nanoparticles with sulfur-doped graphene and with cyclized polyacrylonitrile to provide a robust nanoarchitecture. This hierarchical structure stabilized the solid electrolyte interphase leading to superior reversible capacity of over 1,000 mAh g^−1^ for 2,275 cycles at 2 A g^−1^. Furthermore, the nanoarchitectured design lowered the contact of the electrolyte to the electrode leading to not only high coulombic efficiency of 99.9% but also maintaining high stability even with high electrode loading associated with 3.4 mAh cm^−2^. The excellent performance combined with the simplistic, scalable and non-hazardous approach render the process as a very promising candidate for Li-ion battery technology.

## Abstract (web summary)

Silicon anodes are promising for lithium-ion battery development, but suffer from problems such as undesired volume expansion and solid-electrolyte interface formation. Here, the authors report a hierarchical silicon-sulfur-graphene composite anode which mitigates the problems leading to high performance.

## Graphical abstract

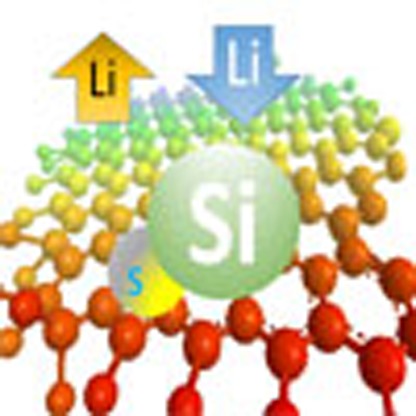

The success of high-performance portable electronics and hybrid (or electric) vehicles strongly depends on further technological progress of commercially available rechargeable batteries^1^. Lithium-ion batteries (LIBs) are considered the most likely energy storage configuration to satisfy these demands^2^,^3^; however, this requires significant advances in terms of power density, energy density, cycle life and safety, as well as lower production costs. Current LIBs systems utilize graphite anodes, where energy is stored by intercalating lithium into the graphite layers. This arrangement, while commercially successful, can only deliver a maximum theoretical capacity of 370 mAh g^−1^ (ref. 4). Incorporating additional components offers the potential to dramatically improve this capacity, whereby silicon can provide up to 4,200 mAh g^−1^ in theory. While Si-based composites offer immense promise as new generation anode materials, extreme changes in volume during lithiation and delithiation lead to structural degradation and debilitating performance loss over time that impedes their practical application^4^,^5^,^6^,^7^,^8^,^9^,^10^,^11^,^12^,^13^,^14^,^15^.

Significant efforts have been devoted to tackling these problems by engineering Si-based electrodes at the nanoscale^5^,^16^,^17^,^18^,^19^. For example, silicon nanoparticles (SiNP) were embedded in a carbon matrix through a multistep process to create nanosized void spaces for accommodating volume changes during lithiation/delithiation^18^. Mesoporous silicon sponges have also been prepared by electrochemical etching of B-doped Si wafers, which were used to minimize the pulverization of silicon. With an additional carbon coating, these materials delivered a capacity of 500 mAh g^−1^ for 1,000 cycles (at a rate of 1 A g^−1^, and an aerial capacity of ca. 1.5 mAh cm^−2^)^20^. Another promising method involved *in situ* polymerization of a conducting hydrogel to coat the SiNP, providing porous space for the large volume expansions^21^. To further improve the performance at a high active material electrode loading, the same group proposed another novel electrode design concept analogous to pomegranates to stabilize the solid electrolyte interface and to provide stable cycling up to 1,000 cycles^20^. Thus, nanostructures materials introduced several solutions to increase the performance of LIB. Nevertheless, new challenges are showing up. The high surface area associated with the very small particles sizes may increase the unnecessary reactions with electrolyte leading to high irreversible capacity. Furthermore, the higher resistance between the particles leads to poor conductivity. It has become necessary to design electrode materials that are structured on microscale, while nanoarchitecture is engineered.

We have introduced the concept of using a flash heat treatment that dramatically improved the interfacial properties in the electrode design^22^,^23^. However, the limitation in electrode loading and the high cost of high temperatures have led us to think of a new electrode design.

Herein we introduce a new electrode design concept that capitalizes on the strong covalent interactions occurring between Si, sulfur, defects and nitrogen. This involves wrapping SiNP with S-doped graphene (SG), and then shielding this composite arrangement with cyclized polyacrylonitrile (PAN). First, we mix SiNP (∼60%), SG, graphitic oxide (GO) and PAN in dimethylformamide (DMF) to form homogenous mixture under ultrasonic radiation. Then, we cast the slurry on Cu current collector followed by drying in convection oven. Finally, the electrodes were cut and pressed, then subjected to a sluggish heat treatment (SHT) by slowly heating in inert gas to 450 °C, then hold for 10 min, followed by furnace cooling. This provided a robust hierarchical nanoarchitecture that stabilized the solid electrolyte interphase (SEI) and resulted in superior reversible capacity of ∼1,033 mAh g^−1^ for 2,275 cycles at 2 A g^−1^. The improved electrode design limited the electrolyte access leading to a high coulombic efficiency of 99.9%, as well as high aerial capacity of 3.4 mAh cm^−2^.

## Results

### Electrode fabrication and treatment

**Figure 1:**
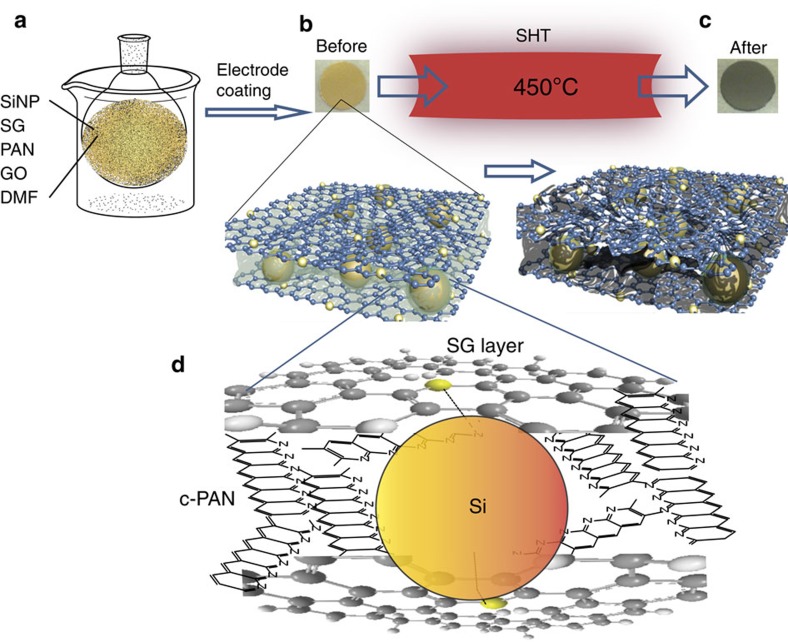
Schematic of electrode process design. (**a**) Components mixing under ultrasonic irradiation, (**b**) an optical image of the as-fabricated electrode made of SiNP, SG and PAN, (**c**) the electrode after SHT, (**d**) Schematic of the atomic scale structure of the electrode.

Mixing of SiNP (∼60%), SG, PAN, GO and DMF was conducted under ultrasonic irradiation (Fig. 1a). This helped achieve homogeneous distribution of electrode components, and possibly allowed preferential attachment of Si to S and defect sites in the SG. Schematic illustration of the SHT of the electrodes, after being coated on copper foils and dried, is shown in Fig. 1b,c. The optical images of the electrode before and after SHT clearly show the colour changes from light to dark, implying the PAN partially carbonizes during the SHT treatment. In this arrangement, we speculate that SiNP are preferentially adsorbed on the sulfur and defect sites in graphene as well as being coated with PAN. As a result, the electrode materials consist of interconnected microparticles. These microparticles are composed of SG nanosheet that sandwich the SiNP clusters, with this entire arrangement encapsulated with PAN. After SHT, PAN is cyclized, tethering the SiNP and SG nanosheet composites together, resulting in a robust structure providing both inner porosity and flexibility.

### Morphology and structure of the electrode

**Figure 2:**
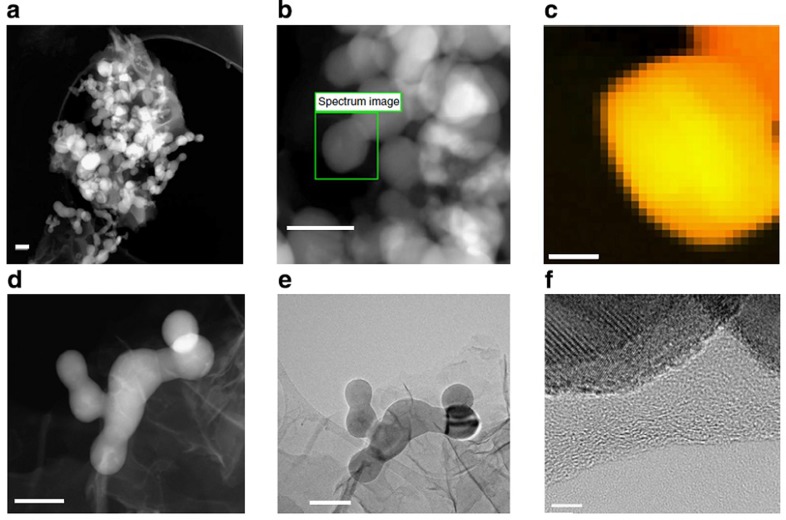
TEM characterization of the electrode. (**a**) HAADF–STEM image of the SG–Si electrode, (**b**) Higher magnification HAADF–STEM image of SG–Si and (**c**) EELS mapping of the elements Si (yellow) and S (red), with each pixel representing 3.4 × 3.4 nm, (**d**) HAADF–STEM images zooming in on interconnected SiNPs in the SG–Si electrode, (**e**) Regular TEM image of the image in (**d**,**f**) HRTEM image of a SiNP with carbon shell and graphene. Scale bars, 100 nm in **a**,**b**,**d**, and **e**, 20 nm in **c**, and 5 nm in **f**.

**Figure 3:**
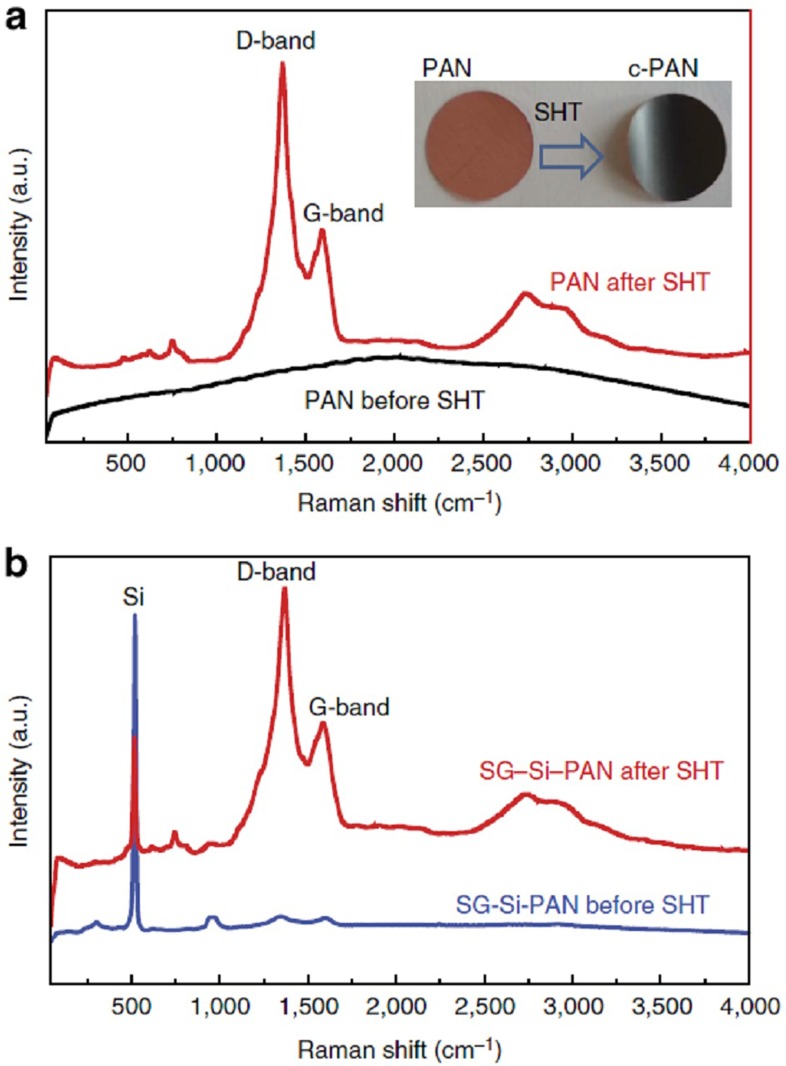
Raman spectroscopy. (**a**) Raman spectra for PAN film on copper before and after SHT (the inset in **a** shows optical image of PAN coated copper foil before and after SHT). (**b**) Raman spectra for SG–Si-PAN electrode surface before and after SHT. a.u., arbitrary unit.

The high-angle annular dark field (HAADF) scanning transmission electron microscope (STEM) image in Fig. 2a shows a micron scale cluster in which the SiNP are well wrapped by SG and invariably dispersed within the nanosheets matrix. Figure 2b displays a higher magnification HAADF–STEM image of the SG–Si electrode, while Fig. 2c displays the corresponding electron energy-loss spectroscopy (EELS) image (red, green, and blue (RGB) mixed colour mapping) of the highlighted area in Fig. 2b. The pixels in the EELS image correspond to 3.4 × 3.4 nm each. The yellow colour is related to Si, while the red colour is sulfur (mixed red and yellow give orange with different degrees relative to the concentration). It can be inferred that sulfur follows the circumference of the SiNP. The corresponding spectrum of the EELS-based elemental mapping is shown in the Supplementary Fig. 1. It, again, confirms the presence of Si, S, N and C, whereby S comes from the SG and N from the cyclized PAN (c-PAN). To show how the binder PAN has shelled the particles and connected them, a zoomed HAADF–STEM, with the corresponding TEM, are presented in Fig. 2d,e, respectively. They clearly show that the particles are interconnected and wrapped with graphene. A closer image of high-resolution transmission electron microscopy (HRTEM) focusing on one particle (Fig. 2f) shows the crystalline Si particles with a shell of c-PAN and graphene nanosheets. Raman spectra of a PAN film deposited on copper foil, then dried, before and after SHT is shown in Fig. 3a. While no features appear before SHT, two characteristic peaks at ∼1,346 and ∼1,605 cm^−1^ are observed after SHT. These peaks correspond to the ‘D' and ‘G' bands from the structural defects and disorder from sp^3^-carbon atoms and the plane vibration of the sp^2^-carbon atoms in two-dimensional lattice of the c-PAN, respectively. This result again confirms that the cyclization of PAN is associated with graphitized carbon. The same features appeared with the electrode materials after subjecting them to SHT (Fig. 3b).

It is well established that sluggish heating can cyclize PAN, whereby c-PAN can provide stabilization of electrode structures^24^,^25^. A small proportion of GO, ∼1%, was added as oxidizing agent to promote cyclization of PAN. The characteristic exothermic peak for PAN cyclization is shown by differential scanning calorimetry in the Supplementary Fig. 2a, with the results consistent with previous reports^26^,^27^. On treatment, PAN loses about 20% of its mass as shown by thermal gravimetric analysis (TGA), with results provided in Supplementary Fig. 2b. The SHT treatment has modified the chemical structure of the PAN causing cyclization. The cyclization process is associated with changes in the nature of chemical binding of nitrogen with an evidence of enriched pyridinic type nitrogen, as shown by the X-ray photoelectron spectroscopy (XPS) results presented in Supplementary Fig. 2c with a shift of binding energy of nitrogen from 399.88 to 398.38 eV (refs 28, 29). After cyclization, PAN has a π-conjugate structure that is believed to lower the electronic and charge transfer resistances of the electrode, as evidenced by the electrochemical impedance spectroscopy shown in Supplementary Fig. 3. After inspecting the HRTEM images introduced in Fig. 2 and the energy-dispersive X-ray (EDX) mapping in Supplementary Fig. 4, it can be proposed that, almost every SiNP is caged in a carbon shell of c-PAN. It is also clearly observed that there is no agglomeration of SiNP.

### X-ray photoelectron spectroscopy

**Figure 4:**
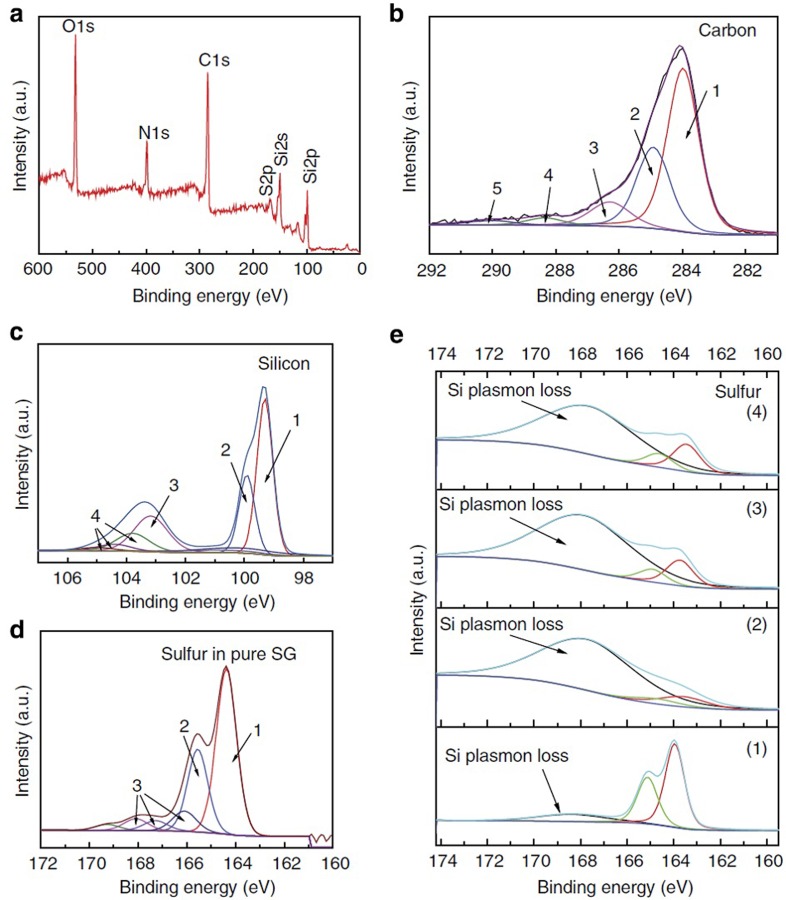
Electrode material characterization for SG–Si. (**a**) XPS survey spectra confirming the elements Si, S, C, N and O, (**b**) high-resolution XPS spectra of carbon in SG–Si, (**c**) high-resolution XPS of Si–2p in SG–Si, (**d**) high-resolution XPS spectra of sulfur in pure SG and (**e**) high-resolution XPS of sulfur in (1) electrode material made of elemental S, SiNP and PAN, (2) electrode material of (1) after being subjected to SHT, (3) electrode material made of SG, SiNP and PAN and (4) electrode material of (3) after being subjected to SHT. a.u., arbitrary unit.

The elemental analysis of the electrode material after being subjected to SHT is determined by the XPS survey spectrum as shown in Fig. 4a, confirming the existence of Si (40%), S (5%), C (40%), N (11%) and O (4%), with all compositions given in atomic %. It should be pointed out that XPS provides high surface sensitivity with analysis depth of about 8–10 nm. Therefore, this elemental quantification is different from the expected values that estimate Si as 60% and S as ∼0.5%. The spectra of C in Fig. 4b shows several common peaks, the first one (1) centred at 284 eV corresponds to sp^2^ hybridized graphitic type carbon. Peak (2), centred at 284.8 eV, denotes the presence of sp^3^ bonded carbon. Finally, peaks (3) and (4) are characteristic of oxygenated carbon and peak (5) is related to Plasmon loss features^30^,^31^,^32^. The core-level spectra in Fig. 4c shows the typical elemental Si peak (1) located at 99.4 eV, with the minor peaks at higher binding energies (∼103.4 eV) related to oxygenated silicon or silicon bonded to sulfur^33^. Figure 4d shows the core-level spectra of S in pure SG, with the atomic % of S of ∼2.5%. The S_2p_ doublet corresponding to the sulfide (C–S–C) structure is observed at 164.0 and 165.2 eV and labelled as (1) and (2). These peak locations are in good agreement with the reported S_2p3/2_ and S_2p1/2_ spin orbit couplet^34^,^35^,^36^. The other minor peaks labelled as (3) in Fig. 4d and located at higher binding energies are attributed to oxygen bound to sulfur^37^. The structure elucidation of SG using XPS were used as the base to determine the basic SG cluster used for DFT calculations discussed *vide infra*. It is important to note that sulfur doped the graphene sheets homogeneously, both on the edges and in the basal planes. This was evidenced by STEM–EDX and EELS mapping shown in Supplementary Fig. 5. A set of samples were prepared as shown below and analysed to understand the covalent chemisorbed interactions that occur between Si and S in SG. The four samples prepared are: (1) Elemental sulfur microparticles, SiNP and PAN dispersed well in DMF, followed by solvent removal; (2) Sample 1 annealed at 450 °C (same as the SHT process); (3) SG+PAN+SiNP, dispersed well in DMF, followed by solvent removal; and (4) Sample 3 annealed at 450 °C (same as the SHT process). High-resolution XPS spectra for all of these samples was obtained and is shown in Fig. 4e. Sample 1 shows the regular S_2p_ orbital split (doublet at 163.98 and 165.08 eV). In addition, a very depressed broad peak is observed at average 168 eV, which may be attributed to silicon loss Plasmon resonance^38^,^39^. Plasmon loss peaks involve a strong probability for loss of a quanta of energy due to electron interaction with the photoelectron^40^. For Sample 2, some sulfur is covalently interacting with silicon, while the majority of sulfur is lost after annealing due to sublimation (melting point ∼120 °C). The XPS results correspondingly show a greatly enhanced peak signal for the silicon-loss Plasmon resonance. SG instead of elemental sulfur was used in Samples 3 and 4. The XPS signals for both these samples also showed a strong peak for silicon-loss Plasmon resonance, indicating possible interactions between the Si and S atoms even before the annealing process. This feature did not change with annealing, indicating a similarly strong interaction between the two elements in both cases. While there is no direct support from literature, here we speculate that the reason of the enhanced Plasmon loss appeared in samples 2–4 is attributed to the interaction of Si with S. While it was not possible to find similar study to show interaction of Si and S, there were few studies that show possible reaction between silicon and sulfur^41^,^42^,^43^,^44^. The morphology investigated by SEM and pore size distribution investigated by Brunauer, Emmett and Teller (BET) were determined for the electrode before and after the SHT process, shown in Supplementary Figs 6 and 7, respectively. The micron sized particles comprising of SiNP dispersed on the sheets of SG and capped with c-PAN are demonstrated. The results of BET analysis also show that the electrode structure developed increased nanoporosity through the SHT process.

### Electrochemical performance

**Figure 5:**
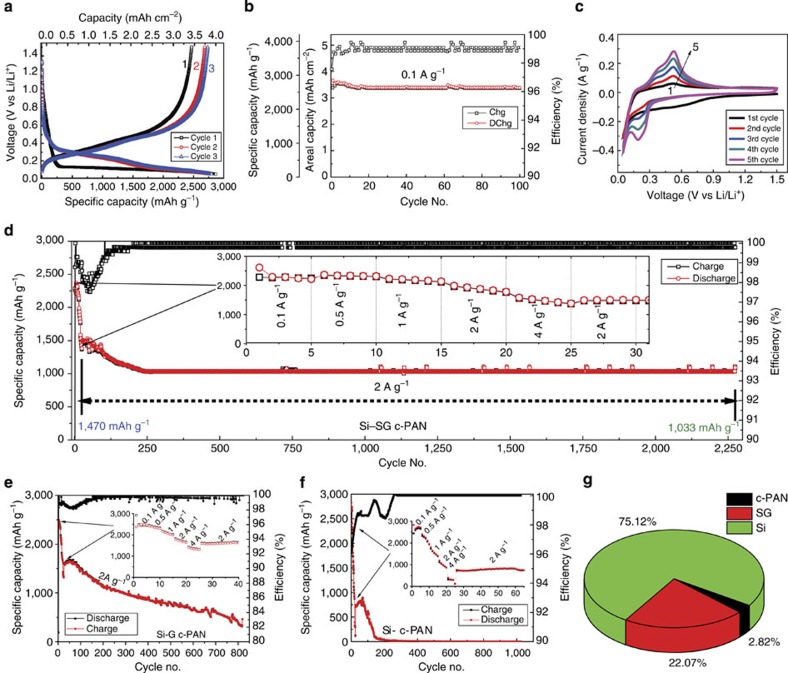
Electrochemical performance of SG–Si. (**a**) Voltage profile of SG–Si anode at 0.1 A g^−1^, (**b**) the corresponding cycle stability, (**c**) cyclic voltammogram curves of the SG–Si coin cell, (**d**) rate capability of SG–Si anode followed by cycle stability at 2 Ag^−1^, (**e**) rate capability of G–Si anode followed by cycle stability at 2 Ag^−1^, (**f**) rate capability of Si–PAN anode followed by cycle stability at 2 Ag^−1^ and (**g**) a pie chart showing the relative contribution of the electrode materials for the capacity seen in **d**.

Figure 5a presents the typical galvanostatic charge/discharge profiles of the SG–Si-based electrode tested at 0.1 A g^−1^ between 1.5 and 0.05 V. The observed plateau in the first discharge curve represents alloying of crystalline silicon with lithium^18^,^45^. The SG–Si delivers an initial discharge capacity of 2,865 mAh g^−1^, based on all masses of SG, c-PAN and Si, with a high first-cycle Coulombic efficiency of 86.2%. If not mentioned, all reported capacities are based on the total mass of SG, c-PAN and Si. The voltage profiles of the subsequent cycles show slightly different behaviour, which is common for the lithiation process of amorphous Si formed during the first cycle. It is noteworthy that the aerial charge capacity is about 3.35 mAh cm^−2^, which is close to the performance targets for next generation high-energy dense LIBs^16^. Figure 5b shows the cycling stability of the SG–Si at 0.1 A g^−1^. A stable cyclability up to 100 cycles can be obtained, with an average capacity of 2,750 mAh g^−1^ (∼3.35 mAh cm^−2^). These results compare favourably to a recently published report^16^. The charge storage behaviour was also characterized by cyclic voltammetry. Figure 5c shows the first five cycles of the SG–Si electrode in a coin cell at a scan rate of 0.05 mV s^−1^. In the cathodic scan, there are two distinctive peaks appearing at 0.27 and 0.22 V versus Li/Li^+^, indicating the formation of Li_12_Si_7_ and Li_15_Si_4_ phases, respectively^46^,^47^. In the anodic direction, the corresponding two peaks are located at 0.31 and 0.49 V, representing the dealloying of Li_*x*_Si to Si. All anodic and cathodic peaks become broader and stronger as a result of cycling, which is a common feature attributed to the conversion of Si into an amorphous phase during lithiation/delithiation. Similar features were observed for a G–Si investigated for comparison as shown in Supplementary Fig. 8. The rate capability of the SG–Si electrode is shown in Fig. 5d, revealing the excellent kinetics of the SG–Si electrode at different currents up to 4 Ag^−1^, Moreover, the robust structure enables a very stable cycling, where a capacity of ca.1,033 mAh g^−1^ can be maintained for 2,275 cycles at a rate of 2 Ag^−1^. By comparison, a similar electrode structure prepared by replacing SG with non-doped graphene gives an inferior rate capability and cycling stability, as shown in Fig. 5e. The high capacity of the G–Si persists only for 80 cycles, then fades gradually, reaching ∼400 mAh g^−1^ after 800 cycles. Such a capacity fading is mainly attributed to the degradation of the Si structure, where the expansion and shrinkage of SiNP during cycling leads to the separation from graphene scaffold, and subsequent loss of conductivity and instability in the SEI structure. The significantly different electrochemical performances put a spotlight on the important role of sulfur in binding the SiNP to the surface of SG, which encouraged us to further investigate it using density functional theory (DFT) calculations discussed below. As a reference, a coin cell made of a SiNP/PAN electrode, fabricated using SiNP and PAN subjected to a SHT, also shows poor rate performance. In addition, its cycle stability persist for only 65 cycles and then degrades rapidly to almost zero capacity (Fig. 5f). These results emphasize the important role of the covalent binding between Si and SG to enable the impressive performance. In all cases, SG–Si, G–Si and even just Si when fabricated using PAN and followed by our SHT treatment persists for at least for 2,275, 80 and 65 cycles, respectively. On the other hand, a coin cell fabricated using the same SiNP (60%), Super P (20%) and the traditional binder polyvinylidene fluoride (PVDF; 20%) without any SHT treatment has degraded very rapidly, as shown in Supplementary Fig. 9. Since we considered the total mass of the electrode during calculation of the capacity, it is important to show the relative contribution of each of the electrode components. Figure 5g is a pie chart showing the relative % contribution of the capacity observed in Fig. 5d. The results are based on the battery performance testing for SG, under similar conditions, which shows average reversible capacity of 235 mAh g^−1^, and an electrode coated with only PAN after SHT treatment, which gave an average capacity of 18 mAh g^−1^ (see Supplementary Fig. 10a and b). To investigate the specific role of c-PAN and SG, reference cells were fabricated from SG–Si–PVDF and GO–Si–PAN, respectively. The battery performance of these two cells decayed rapidly as shown in Supplementary Figs 11 and 12. This emphasizes the synergy of the SG–Si–c-PAN in enhancing the electrode stability and providing stable cycling.

The volumetric capacity for the cell presented in Fig. 5b was calculated and the result was plotted in Supplementary Fig. 13. It reveals that the SG–Si–c-PAN electrode is able to provide a reversible capacity of ∼2,350 mAh cm^−3^ for up to 100 cycles. Coin cells fabricated using different electrode composition of 40:30:30 (Si–SG–PAN) were tested and the results were introduced in Supplementary Fig. 14. It reveals similar trend of stable cycling and improved rate capability.

**Figure 6:**
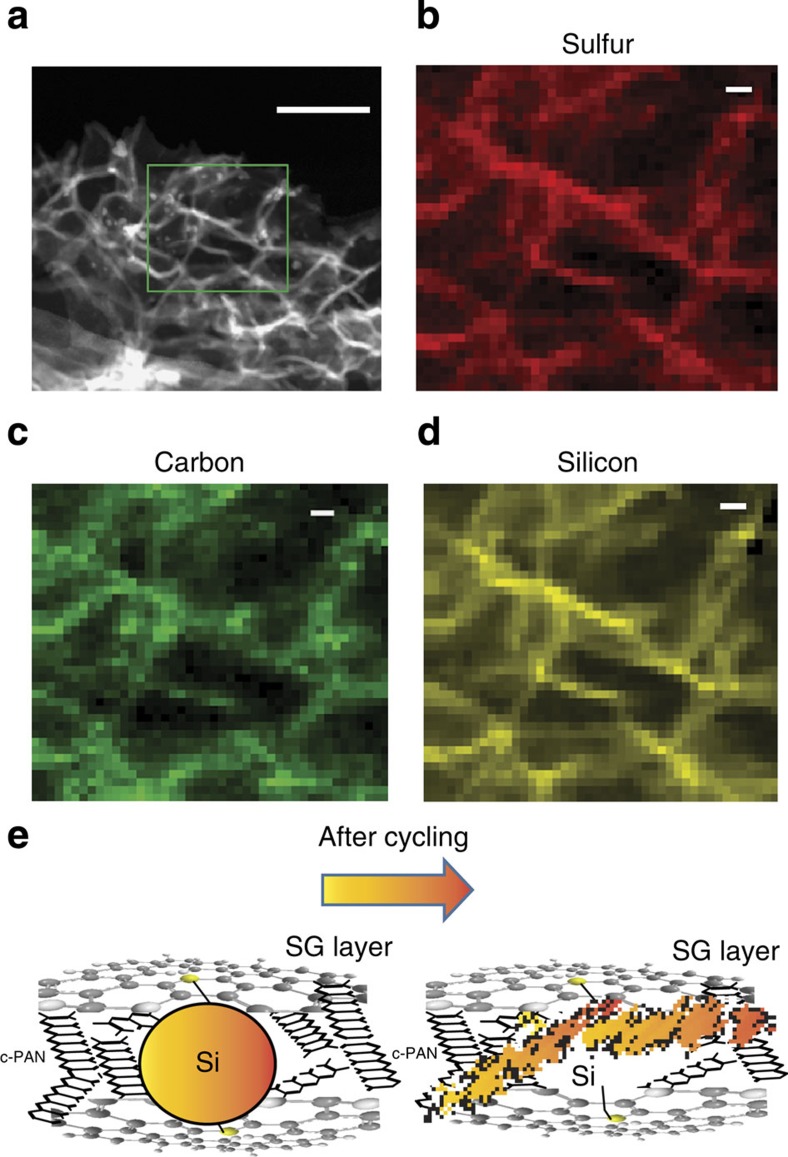
Characterization of SG–Si electrode material after cycling for 2,250 cycles. (**a**) HAADF–STEM image of the SG–Si electrode after cycling, (**b**–**d**) the elements mapping by EELS for the area marked in image. Scale bar, 100 nm in **a** and 10 nm in **b**–**d**. Each pixel in **b**–**d** represents 3.4 × 3.4 nm. (**e**) A schematic representation to explain the structure change in the electrode before and after cycling. Before battery cycling SiNP are dispersed, and bond with S on surface of SG with c-PAN further connect the SiNP with SG. After battery cycling, the SiNP change to amorphous structure and spread and confine in the crinkles of SG.

After cycling a coin cell for 2,275 cycles (Fig. 5d), the cell was disassembled and the SG–Si electrode was subjected to further characterization. Figure 6a shows a HAADF–STEM image of the electrode structure and Fig. 6b–d provide the corresponding coloured EELS mapping for the elements S, C and Si, respectively (each pixel is 3.4 × 3.4 nm). This characterization shows that the Si, as a result of frequent cycling, confine in the wrinkles of SG, and capped with c-PAN, utilizing the covalent interaction between Si, SG and N. The location of the SiNP is associated with regions of high sulfur and carbon. It is clear that the engineered nanoarchitecture of the electrode design along with the covalent interaction occurring between Si an SG, prevented agglomeration of Si and maintained stable reversible cycle stability for 2,275 cycles. The same electrode was mapped using EDX for comparison and the results was presented in Supplementary Fig. 15. It is important to emphasize here that EELS provides a near atomic scale resolution to depict the distribution of atoms throughout the sample. EELS also has a high sensitivity for lighter elements, explaining why the signals from both carbon and sulfur are clearly distinguished. Figure 6e presents conceptual design of the electrode structure before and after frequent cycles of continues lithiation/delithiation. On the other hand, inspection of the electrode of the cell based on G–Si–c-PAN after being cycled under the same conditions shown in Fig. 5e by STEM reveals that by continuous cycling silicon reveals more agglomeration, Supplementary Fig. 16. This emphasizes the important role of SG, which prevents agglomeration of silicon and maintains electrode stability over a large number of cycles.

### DFT calculations

In the present study, the graphene surface was modelled using a hydrogenated graphene cluster (C_54_H_18_), which is also referred as H-passivated graphene (Supplementary Fig. 17). The optimized bonding distances of C–C (1.42 Å) and C–H (1.09 Å) in this model are in good agreement with that for bulk graphite^48^. Based on this H-passivated C_54_H_18_ cluster, and based on bonding configuration elucidated by XPS presented in Fig. 4d, a structure of SG is proposed. The optimized SG structure with some key structural parameters is shown in Supplementary Fig. 18. It can be seen that the SG has a distorted configuration. In all the calculations, all the atoms in the cluster were allowed to relax.

To describe the interactions between the Si and graphene, the bonding energies (BE) of Si were defined by equation (1):


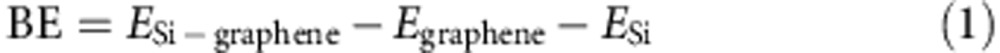


where *E*_Si−graphene_, *E*_Si_ and *E*_graphene_ represent the energies of the Si bound to the graphene structure, the Si atom and the graphene structure, respectively.

**Figure 7:**
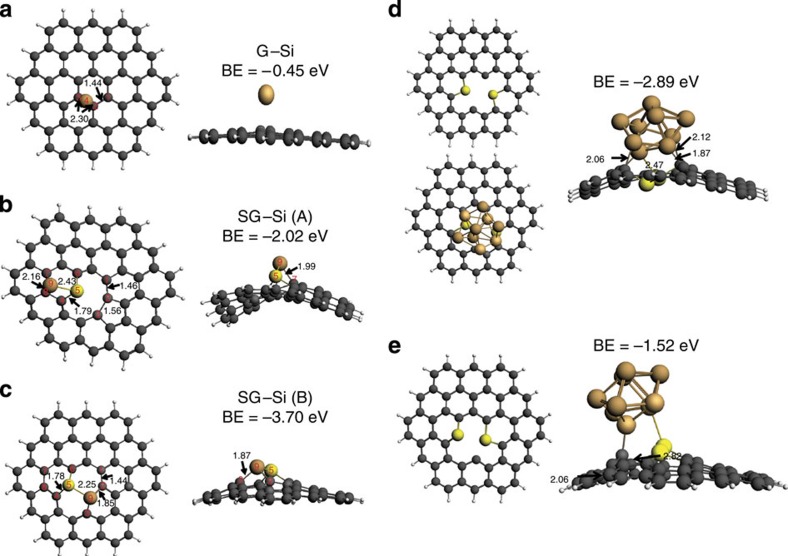
DFT quantum calculations for G–Si and SG–Si systems. Geometries and binding energy (BE) of the stable Si adsorption configurations on (**a**) graphene, referred as G–Si; (**b**,**c**) on sulfur-doped graphene, referred as SG–Si(A) and SG–Si(B), respectively, C atoms are coloured grey, H atoms white, S atom yellow, Si atom brown. Some of the important atoms were labelled, and they correspond to the atoms in Table 1, and (**d**,**e**) The DFT calculated BE of the stable cluster of nine Si atoms' adsorption configurations to SG with different defect configurations. The bond lengths shown in the figure are in angestroms.

Si adsorption on different sites of the SG was studied. The results are compared with those obtained on undoped graphene. Figure 7a presents the configuration of the stable Si adsorption on graphene (G–Si), with Si sitting at the bridge site with adsorption energy of 0.45 eV. Two stable configurations for Si adsorption on SG were observed. The first is represented as SG–Si(A), which reveal the bonding of Si to location (A), Fig. 7b. The second represents binding to location (B) and represented as SG–Si(B) (Fig. 7c). In SG–Si(A), Si was found to bind to S and two ‘saturated' C atoms (C_7_ and C_8_ ), with the corresponding binding energy of −2.02 eV. On the other hand, at the second position, SG–Si(B), Si binds to S and two C's at the defect sites (C_2_ and C_3_) forming two Si–C and one Si–S bonds, leading to a binding energy of −3.70 eV. The higher binding energy in the latter case indicates Si would be more energetically favourable to bind to the defect C_2_ and C_3_ atoms. Most importantly, the results show that Si attached on SG structure has a much higher binding energy than that on graphene (G–Si). This result introduces a strong explanation for the much longer cycle stability in SG–Si than in G–Si. The binding energy of silicon cluster made of nine silicon atoms to different defect configuration in SG (Fig. 7d,e) was also studied. As expected, the covalent interaction occurs between only two of the silicon atoms in the cluster adjacent to the S and defect in SG. The binding energy was found to be dependent on the defect configuration. Supplementary Figure 19 shows the binding configuration with smaller cluster of four Si atoms. The same cluster binds to SG stronger than binding to defect-free graphene.

Hirshfeld charge analysis was also conducted to evaluate the stability of Si on G and SG. The calculated charge distribution before and after the Si adsorption on G and SG are given in Table 1. The results show that Si has a positive charge after its adsorption on G and SG, which indicates that there are electrons flow from the Si atom to the graphene substrate on Si adsorption. However, the electron flow is more significant for Si adsorption on SG than that on G, because Si deposited on SG has a larger positive charge than that on G. Table 1 also shows that the C atoms that are bonded with the Si atom in SG–Si, such as C_7_ and C_8_ in SG–Si(A), C_2_ and C_3_ in SG–Si(B), have more negative charges than in G–Si (C_2_ and C_3_). All these observations indicate that the bonding between Si and SG is stronger than that on G, providing further support for the stability of Si on SG.

**Figure 8:**
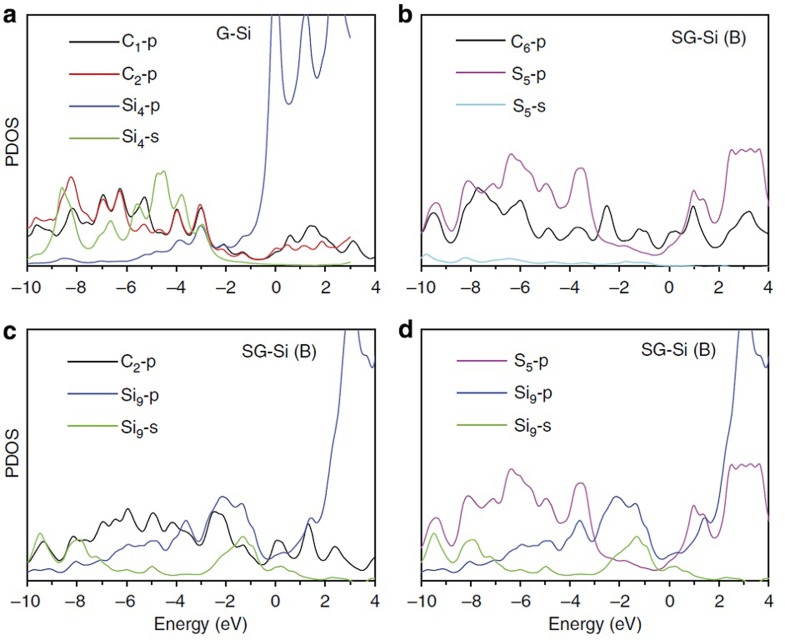
Projected density of states (PDOS). The PDOS for Si atom and the individual C atoms involved in (**a**) Si adsorption on graphene, G–Si, and (**b**–**d**) Si adsorption on sulfur-doped graphene, SG–Si(B).

To better understand the covalent synergy between Si and graphene substrates, the projected density of states of the Si atom over G and SG were calculated, based on the electron structure and bonding. As shown in Fig. 8a, there is a harmonic 2p–2p overlaps between the C_1_–2p and C_2_–2p states at the whole energy level (from 0 to −10 eV) in SG, showing the strong interaction between the two C atoms. However, for Si and C, the harmonic overlap occurs only between Si_4_–2p and C_2_–2p at a narrow energy level (−2 to −4 eV), indicating a weak interaction between Si_4_ and C_1_ atom. For. SG–Si(B), a large overlap between the C_6_–2p and S_5_–2p state was observed (Fig. 8b), indicating a strong S–C bonding. Figure 8c shows that, more Si_9_–2p state is occupied in SG–Si(B) and well mixed with C_2_–2p state at a much broader energy level (from −1 to −9 eV) as compared with that in G–Si. In addition, there is also a harmonic overlap between Si_4_–2p and S_5_–2p state (Fig. 8d). The analysis of the projected density of states revealed that the covalent synergy was mainly due to the mixing between the C–2p and Si–2p states, and the C_2_–Si_9_ bond is much stronger than the C_2_–Si_4_ bonding in G–Si, which attributes to the significantly improved cycle stability.

The mobility of the adsorbed Li atom was also studied. Supplementary Figure 20 shows the transition state along the diffusion pathway. It was found that, for Li atom that diffuses away from the aforementioned most stable sites in G–Si, it needs to overcome an energy barrier of 0.75 eV, as shown in Supplementary Fig. 20a. However, the study of Li surface diffusion on SG–Si(B) cluster shows that Li diffusion proceeds with a barrier of 0.53 eV (Supplementary Fig. 20b), which is slightly lower than that found on G–Si. This observation indicates that SG could boost the mobility for Li atoms on Si–SG interface, which facilitate the charge transfer.

## Discussion

According to the results presented above, we ascribe the elegant cycling stability and improved rate capability to the robust, nanoarchitectured and structurally stable electrode design. This capitalizes on the changes occurred during electrode processing. During the SHT process, several changes to the electrode structure are proposed: (1) PAN is cyclized by forming graphitized carbon with six-membered ring structure hosting the nitrogen atoms in pyridine-like assembly. (2) Silicon anchor and covalently interacts with the sulfur atoms, the activated carbon associated with nanoholes in SG and nitrogen in the c-PAN. (3) The reconstruction and atomic scale architecturing of the electrode lead to a robust structure in which the SiNP are protected by scaffold of graphene nanosheets and a web of c-PAN. The c-PAN forms an effective shielding around the SiNP, which are already anchored on SG through covalent interactions as confirmed by DFT calculations. In addition, c-PAN sticks between the SG nanosheets, providing a three-dimensional (3-D), interconnected structure that enables enhanced conductivity and material robustness, as shown schematically in Fig. 1d.

It can be noted that the SiNP, after 2,275 repetitive expansion and contraction cycles, are fractured and pulverized into smaller particles. However, those fractured Si particles are still confined within the continuous channels of the c-PAN shell, which is overlaid on SG and maintains the electrical connection between Si and graphene. The synergy of the interactions among Si/SG/c-PAN leads to excellent cycle efficiency and capacity retention. The unique and elegant special arrangement in the 3-D structure of the electrode provided critically sized voids along with elasticity that accommodated repetitive volume expansion and contraction. This resulted in preserving electrode integrity and prevented degradation. Furthermore, sandwiching SiNP that have been capped with cyclized PAN, between SG nanosheets forms laminated structure with limited open channels. This supress the penetration of the electrolyte into the bulk of the electrode and limited most of the SEI formation to the surface. We believe that the TEM (EELS) images shown in Fig. 6 can provide some indirect evidence that most of the SEI formed on the outside. The surroundings of Si is quiet clean. If the SEI formed on SiNP, we should be able to see large amount of SEI covering Si, since it is difficult for the fractured SEI to come out. Another possibility is that the SEI would preferentially formed on the defective areas in the graphene, which might prevent solvent getting into the space inside. Here we were trying to emphasis that most of SEI formed on graphene surface, which is more stable comparing with those formed on Si surface.

Based on our DFT model, the Si atom has covalent interactions with a sulfur atom in SG and two adjacent carbon atoms. The equivalent strength of this covalent interaction is similar to that of a single covalent bond. This interaction may not involve the Si atom reacting directly with sulfur to form either SiS or SiS_2_, as this would require debonding of sulfur from within the graphene matrix, and may result in electrode degradation. In the case of Si clusters (to simulate nanoparticles), only a small portion of the silicon atoms form this covalent interaction with the SG. We believe that this type of Si does not participate in alloy formation with lithium; however, provides an anchoring site for the majority of Si atoms within the nanoparticle that are readily available for alloying/dealloying, thereby contributing to the observed capacity.

It can be seen that Si binds more strongly to SG than on G. One reason is the covalent interaction of Si atoms with the sulfur atom. The second reason is because the increased charge density on the defective (with nanoholes) carbon adjacent to sulfur. This indicates a covalent synergy for the interaction between Si and SG leading to a superior material electrochemical performance, which has not been seen with Si–G. It is clearly shown that, even after 2,275 cycles of charge/discharge, the amorphous SiNP re-organized into channels of the cyclized PAN and the sulfur pathway on graphene, as seen in Fig. 6.

In summary, the novel design of a Si-based electrode through the covalent binding of commercial SiNP and SG along with cyclized PAN offers exceptional potential in the practical utilization of Si anodes for LIB technologies. This covalent synergy enables superior cycling stability along with a high aerial capacity of the electrode, which is close to that of commercial technologies. Such a rational design and scalable fabrication paves the way for the real application of Si anodes in high-performance LIBs. The interaction between S and Si plays a critical role of improving the long-term cycle stability, in addition, the synergistic effect of the covalent bonds between Si–S, the facilitated charge transfer by 3-D graphene network and cyclized PAN and the improved electrode integrity all contributed to the superior cycle performance.

## Methods

### Preparation of SG

100 mg of GO prepared by a modified Hummer's method^35^,^36^,^49^ was mixed with 100 mg of phenyl disulfide by grinding. The materials were loaded into a tube furnace and kept outside the heating zone until the furnace temperature reached 1,000 °C. The sample was then slid into the heating zone where it remained for 30 min under argon protection, followed by cooling to room temperature. Graphene was prepared under identical conditions without phenyl disulfide.

### Electrode fabrication and testing

Electrodes were fabricated using commercially available (Nanostructured & Amorphous Materials, Inc., Houston, USA) SiNP with a size range of 50–70 nm. A slurry consisting of 60 wt% SiNP, 20 wt% PAN, 19 wt% SG and 1 wt% GO was prepared in DMF.The addition of GO was to induce cyclization of PAN by oxidation. The slurry is mixed under alternating magnetic stirring and ultrasonic radiation (1 h each, for three times). The slurry was then coated on Cu foil, dried in a convection oven at 353 K for 1 h, and then in a vacuum oven at 363 K overnight. Circular working electrodes of 1 cm^2^ were cut with the average mass loading of silicon on the electrodes ranging from 0.8 to 1.5 mg cm^−2^. The electrodes were then subjected to the SHT process. They were placed into a quartz tube of a horizontal tube furnace, then subjected to slow heating up to 723 K (450 °C) in 2 h, then holding for 10 min and then for furnace cooling (almost in another 2 h). The treatment was performed under Argon gas flow of 100 standard cubic centimeter per minute (SCCM).

Coin type half cells were fabricated in an argon-filled glove box with the working electrode and a Li metal counter electrode. The electrolyte used was 1 M LiPF_6_ in 30 wt% ethylene carbonate, 60 wt% dimethyl carbonate, and 10 wt% fluorinated ethylene carbonate. Galvanostatic charge/discharge testing was carried out at a cutoff voltage range of 0.05–1.5 V with different current densities for rate capability testing. Cyclic voltammetry, at a scan rate of 0.05 mV s^−1^ between 1.5 and 0.05 V, was conducted using a Princeton Applied Research VersaSTAT MC Potentiostat. One reference coin cell electrode was prepared with the same composition as above, except for the SG, was replaced with graphene. Another reference electrode was fabricated using the ratio of 70 wt% SiNP, 30% PAN as a binder. These electrodes were subjected to SHT treatment.

### Material characterization

The morphologies of the electrode material were imaged using a TEM (JEOL 2010F TEM/STEM field emission microscope) equipped with a large solid angle for high X-ray throughput, and a Gatan imaging filter for energy filtered imaging. TGA and differential scanning calorimetry were measured using TA instrument Q500. The TGA testing was performed in air with a temperature range of 25–850 °C and a ramp rate of 10 °C min^−1^. Raman spectroscopy were recorded using Bruker Senterra device, applying laser with a wavelength of 532 nm.

### Quantum mechanics computational method

The DFT calculations were carried out using the Amestrdam Density Functional^50^,^51^. The electron wave functions were developed on a basis set of numerical atomic orbitals and of Slater-type orbitals. In addition, the triple polarization basis of Slater-type orbitals was utilized. We used PBE−D3 to perform the calculations^52^ where the generalized gradient approximation for the exchange and correlation energy terms is used. This explicitly takes into account the dispersion correction. This is a widely used function for catalysis applications and can produce reliable energetics on graphene systems^53^,^54^.

## Additional information

**How to cite this article:** Hassan, F. M. *et al*. Evidence of covalent synergy in silicon–sulfur–graphene yielding highly efficient and long-life lithium-ion batteries. *Nat. Commun.* 6:8597 doi: 10.1038/ncomms9597 (2015).

## Supplementary Material

Supplementary Information Supplementary Figures 1-20

## Figures and Tables

**Table 1:** Hirshfeld charges distribution before and after Si adsorption.

**Atoms**	**Si adsorption on G**		**Si adsorption on SG**		
	**G**	**G–Si**	**SG**	**SG–Si(A)**	**SG–Si(B)**
C_1_	−0.001	−0.004	0.010	−0.006	−0.004
C_2_	−0.001	−0.028	0.003	−0.022	−0.113
C_3_	−0.001	−0.029	0.004	−0.013	−0.100
C_4_ (or Si_4_)		0.120	−0.016	−0.001	−0.019
S_5_			0.093	0.214	0.206
C_6_			−0.016	−0.035	−0.024
C_7_			−0.003	−0.070	−0.013
C_8_			−0.009	−0.028	−0.006
Si_9_				0.190	0.145

The charge was calculated for the indicated atoms on graphene (G) and sulfur-doped graphene (SG), atoms labelling are indicated in Supplementary Figs 9 and 10.
