# Supplementary material for: Evidence of covalent synergy in silicon–sulfur–graphene yielding highly efficient and long-life lithium-ion batteries
Source: Nat Commun. 2015 Oct 26;6:8597. doi: 10.1038/ncomms9597 (PMC4639807; doi:10.1038/ncomms9597)
Supplement: Supplementary Information — Supplementary Figures 1-20 [file ncomms9597-s1.pdf]

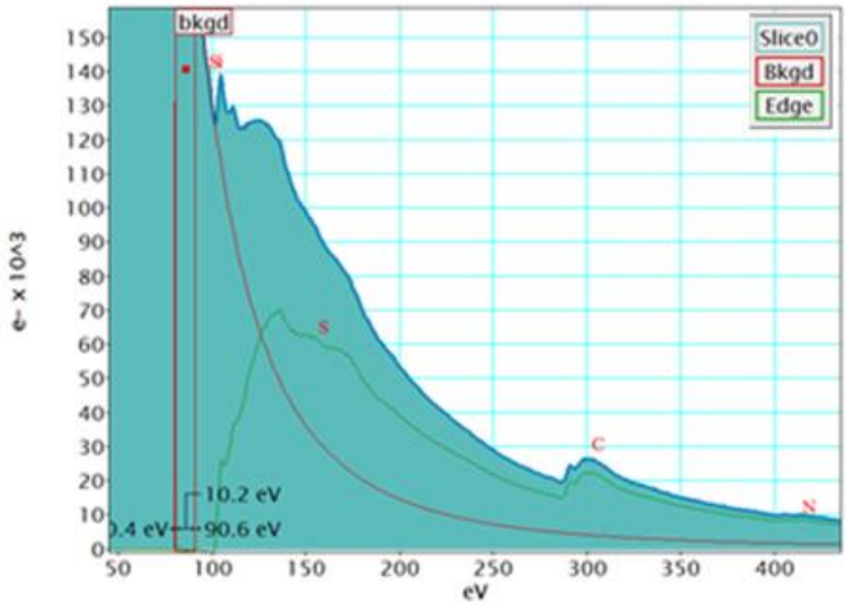

Supplementary Figure 1. **Element analysis in electrode**. Electron energy loss spectrum for SG-Si electrode after sluggish heat treatment (SHT).

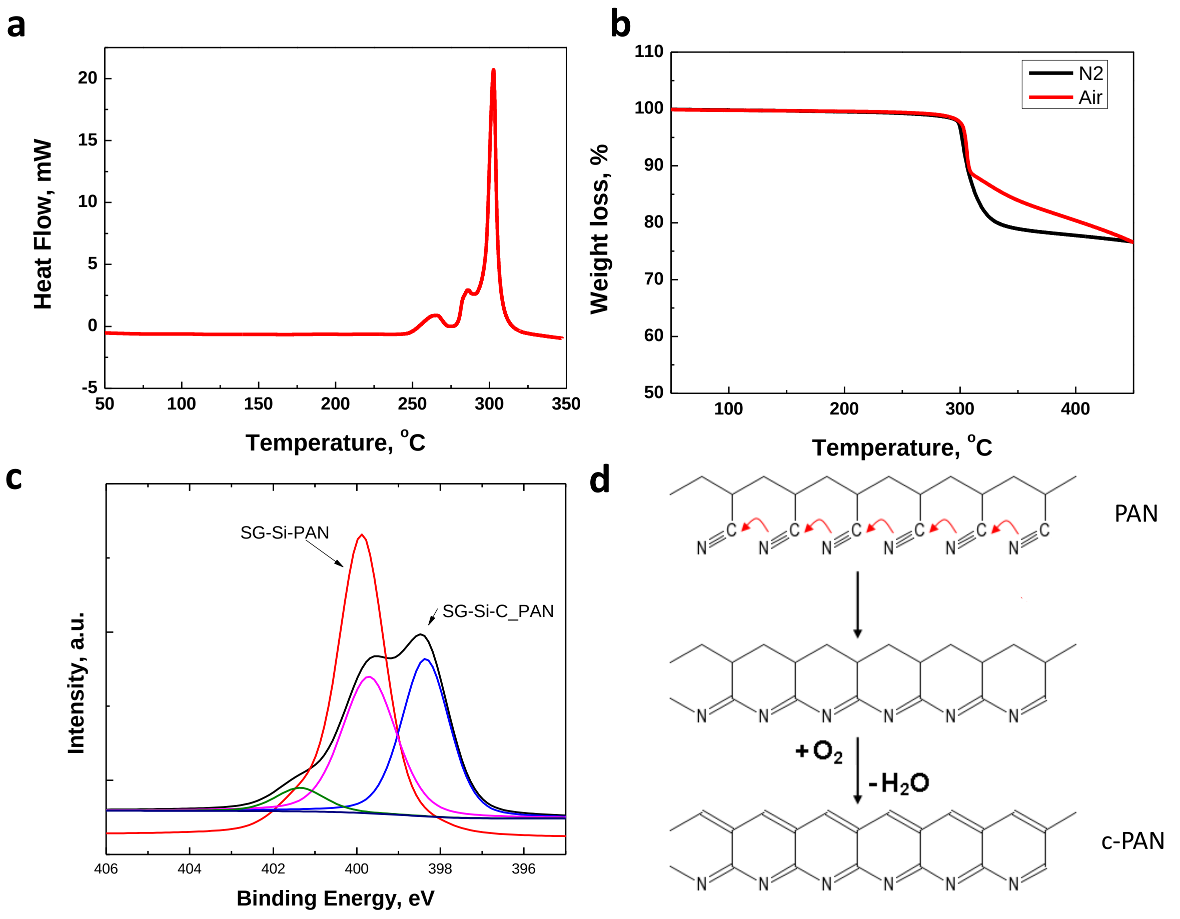

Supplementary Figure 2. **Structure changes of PAN before and after SHT**. (a) Differential scanning calorimetry (DSC) for polyacrylonitrile (PAN) in nitrogen showing a characteristic peak at ~ 300°C, which corresponds to PAN cyclization as proposed in (d); (b) Thermogravimetric analysis for PAN in both air and in nitrogen. During cyclization in nitrogen there is more loss in mass which reveals it is more efficient than in air. By cyclization PAN loses ~ 20% of its mass, (c) Nitrogen high resolution XPS of SG-Si-PAN (before SHT), and SG-Si-C_PAN (after SHT)

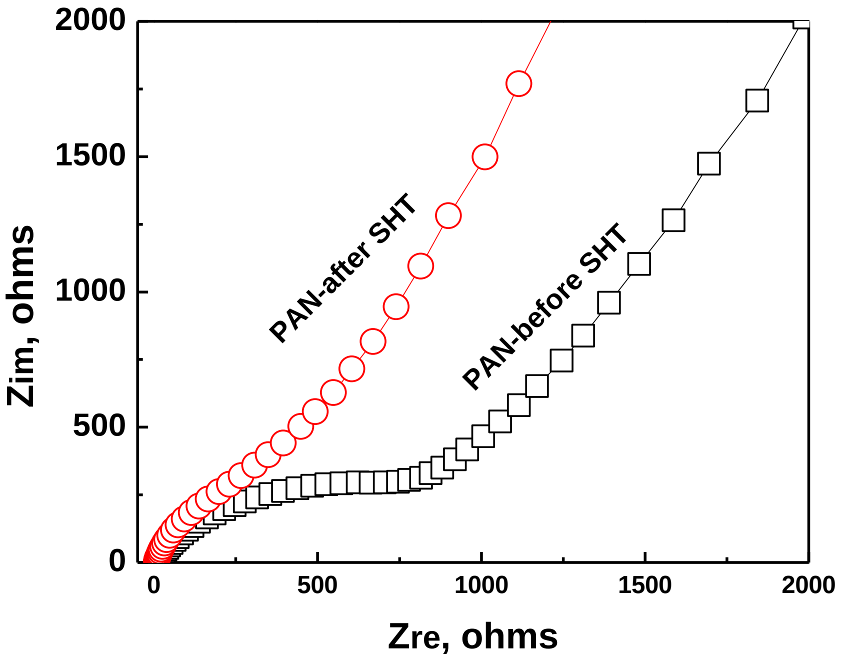

Supplementary Figure 3. **Effect of annealing PAN on resistance.** Electrochemical impedance for a coin cell fabricated using PAN-coated copper foil vs lithium, same method of cell testing as described in the experimental section. The figure reveals that both the electrode series resistance and the charge transfer resistance have been decreased after the sluggish heat treatment.

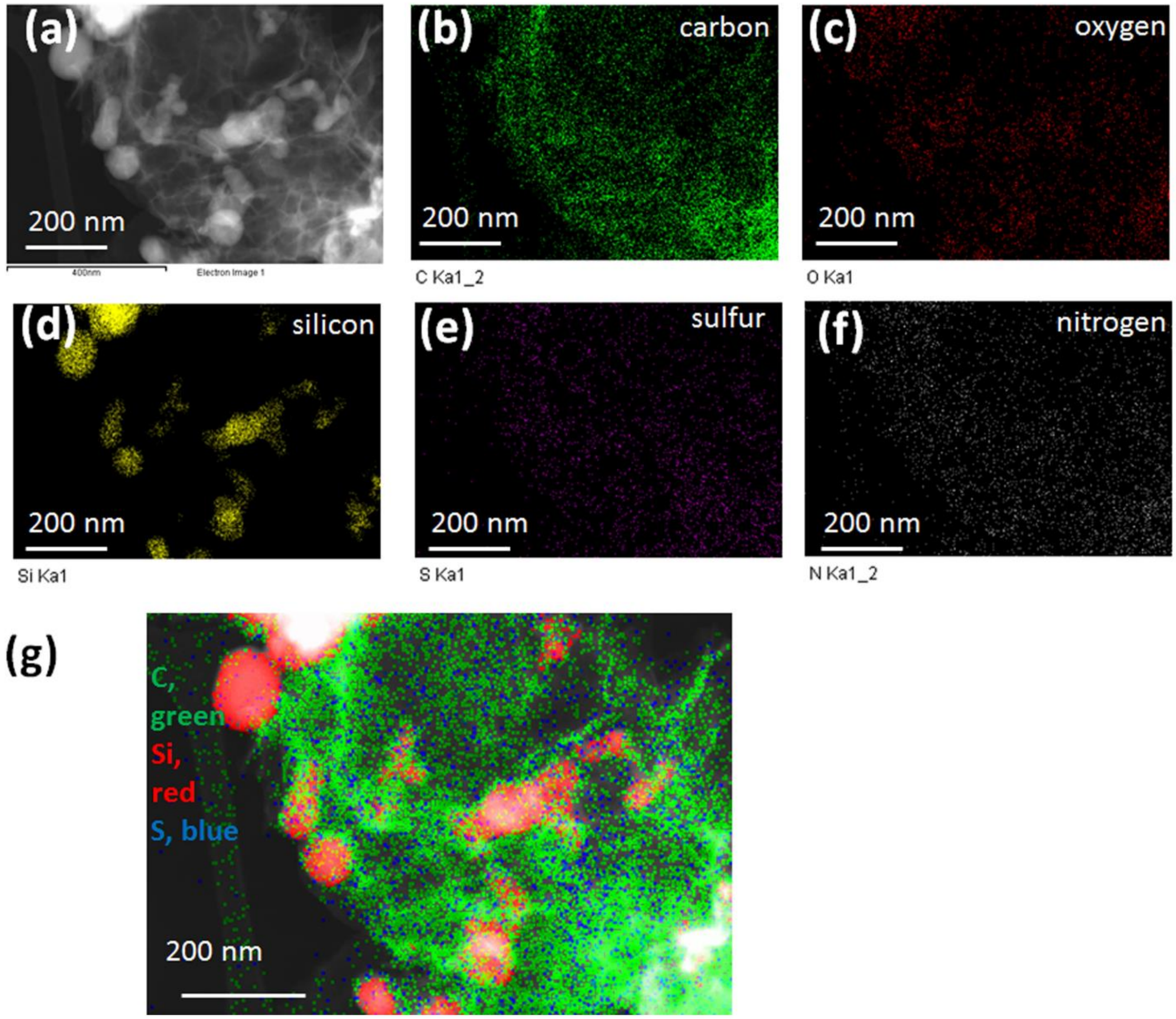

Supplementary Figure 4. **Morphology of the electrode**. (a) TEM image of SG-Si electrode material, (b-f) the corresponding EDX mapping of the elements carbon, oxygen, silicon, sulfur, and nitrogen, respectively, and (g) overlaid colour map of carbon (green), silicon (red), and sulfur (blue).

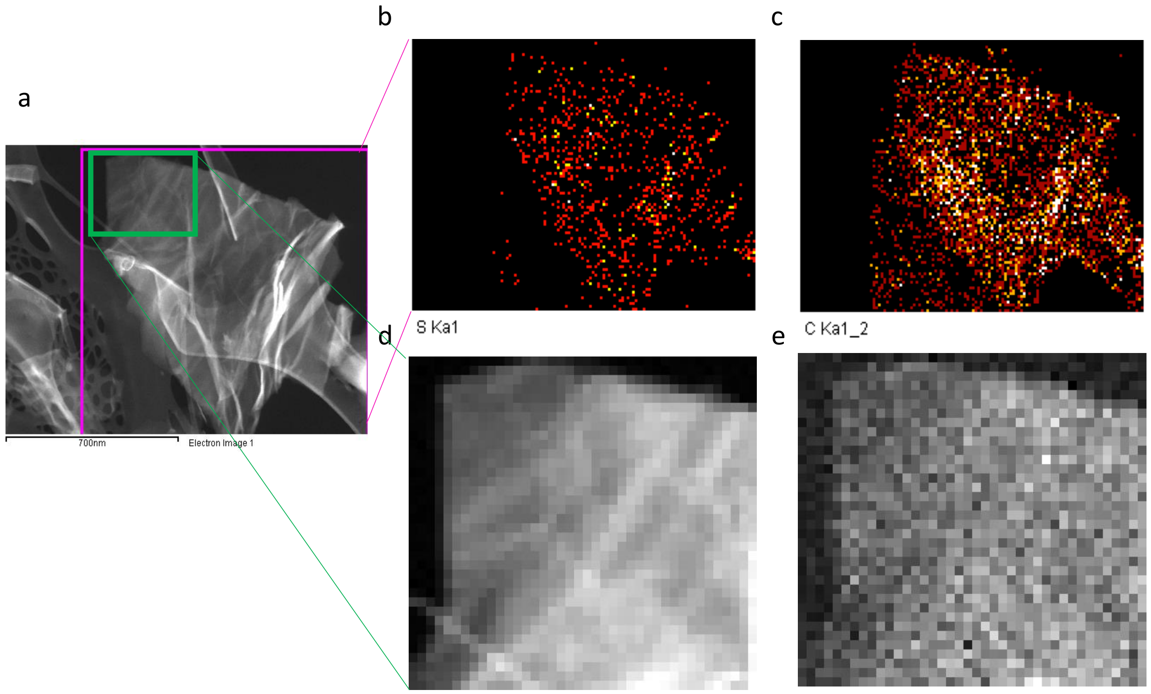

Supplementary Figure 5. **Sulfur distribution on SG nanosheet**. (a) STEM-HAADF of a SG nanosheet in a micron size, (b) and (c) are the EDX mapping for sulfur and carbon, respectively; (d) is the electron energy loss spectroscopy (EELS) mapping and € represent the EELS mapping of sulfur in pixilated grey color, each pixel represent 10 x 10 nm. The figure clearly prove the doping with sulfur in the bulk of SG nanosheet as well as on the edges.

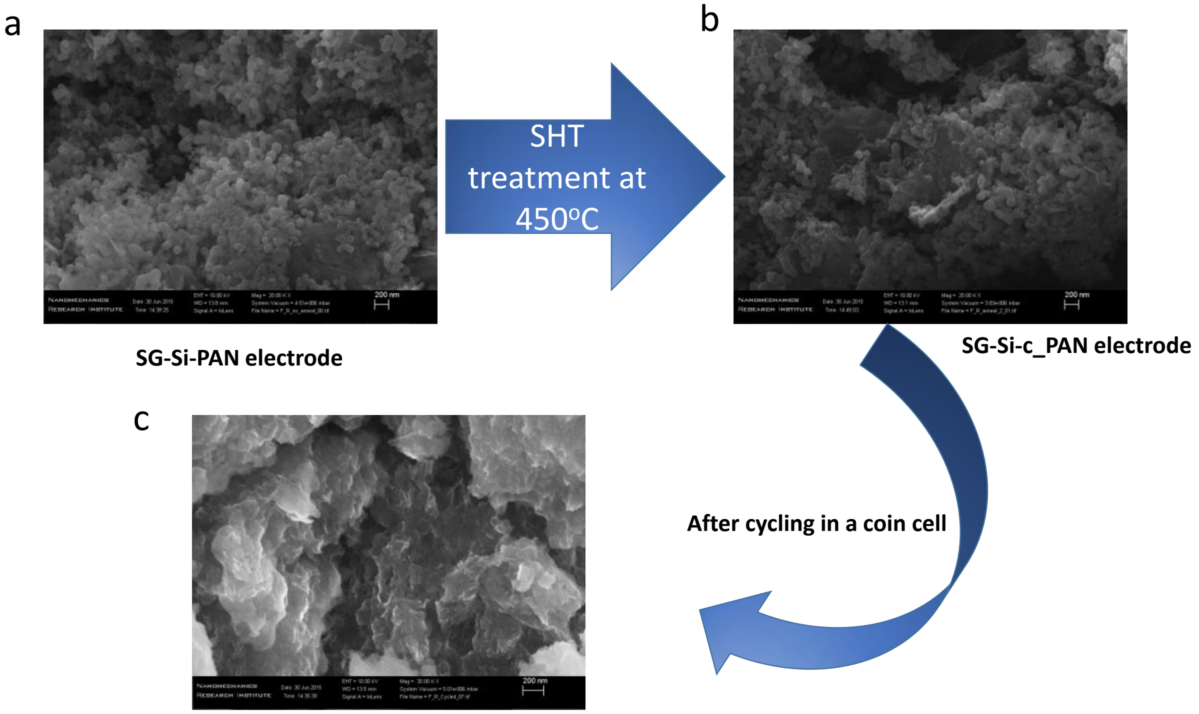

Supplementary Figure 6. **Morphology of SG-Si-PAN electrode**. (a) The as-prepared electrode after drying, (b) The electrode after sluggish heat treatment, and (c) The electrode extracted from a coin cell which was cycled for 100 cycles.

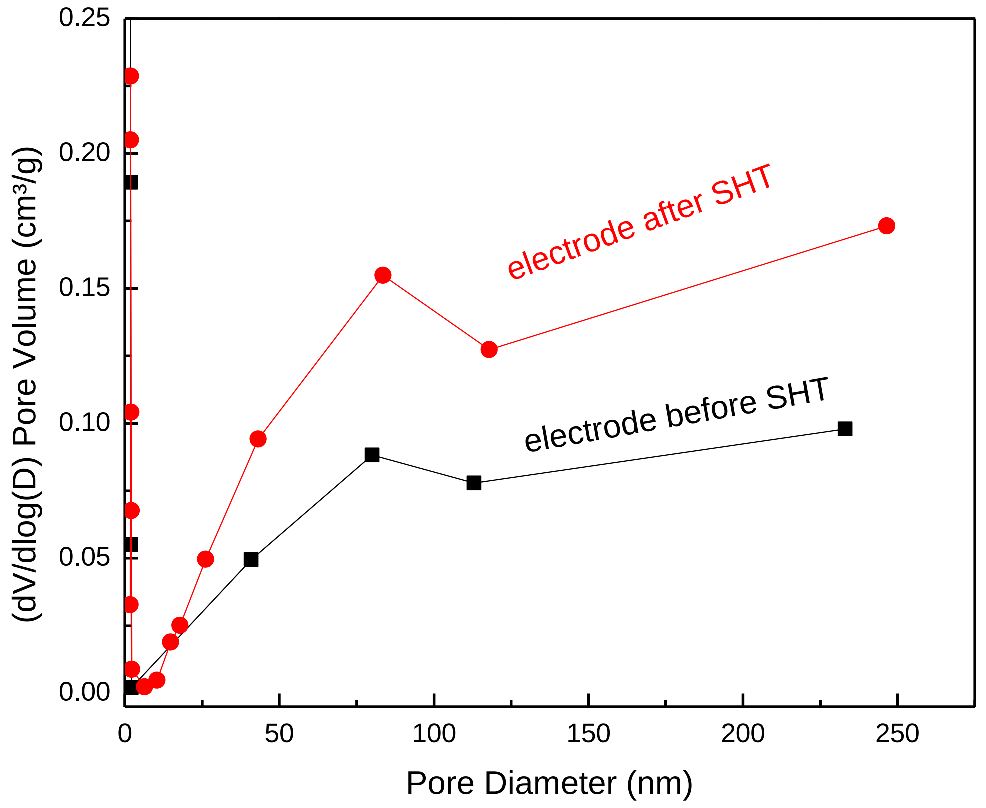

Supplementary Figure 7. Porosity in the electrode. Comparison of pore size distribution for the SG-Si electrode before and after SHT**.** The pore volume increases after SHT which provide void space that compensate the volume expansion of Si during lithiation.

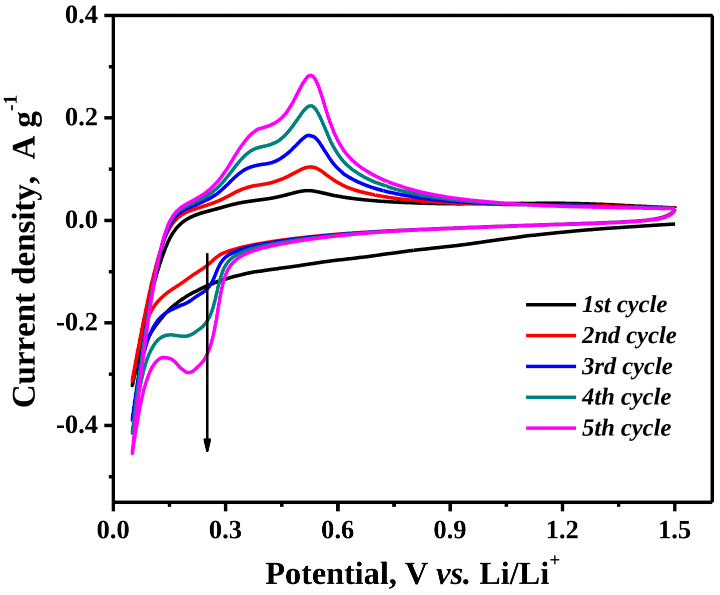

Supplementary Figure 8. **Cyclic voltammogram curves of G-Si anode material in coin cell**.

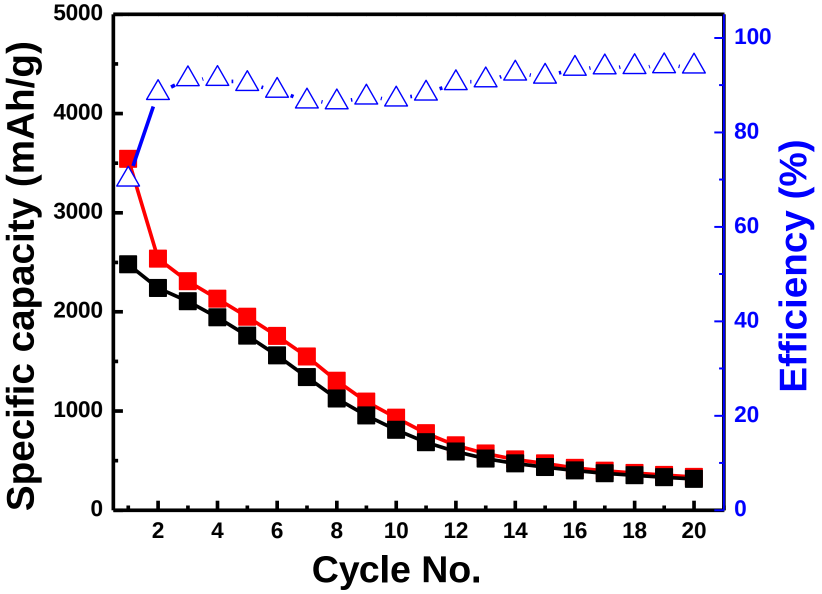

Supplementary Figure 9. **Cycle stability of a reference cell.** The cell was fabricated using SiNP (60%), PVDF (20%) as binder, and super P (20%) as conducting carbon, the performance was tested at 0.1Ag^{-1}.

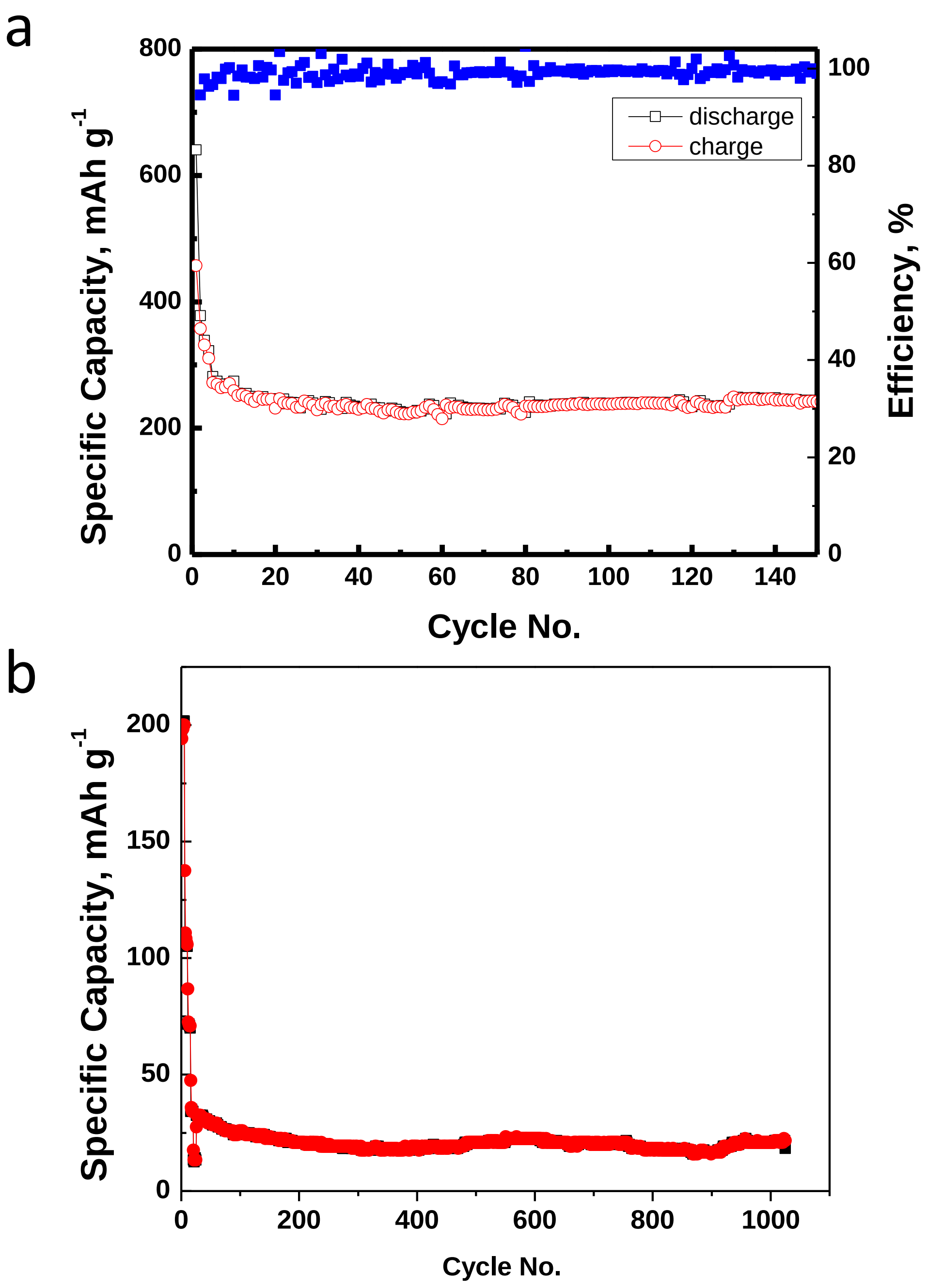

Supplementary Figure 10. **Reference battery testing**. (a) SG-PAN, and (b) only c-PAN, after being subjected to SHT treatment. The cells were tested at 0.1A g^{-1} then continued at 2 A g^{-1}. The SG-PAN provide reversible capacity of ~ 250 mAh g^{-1} and the c-PAN provide ~ 25 mAh g^{-1}.

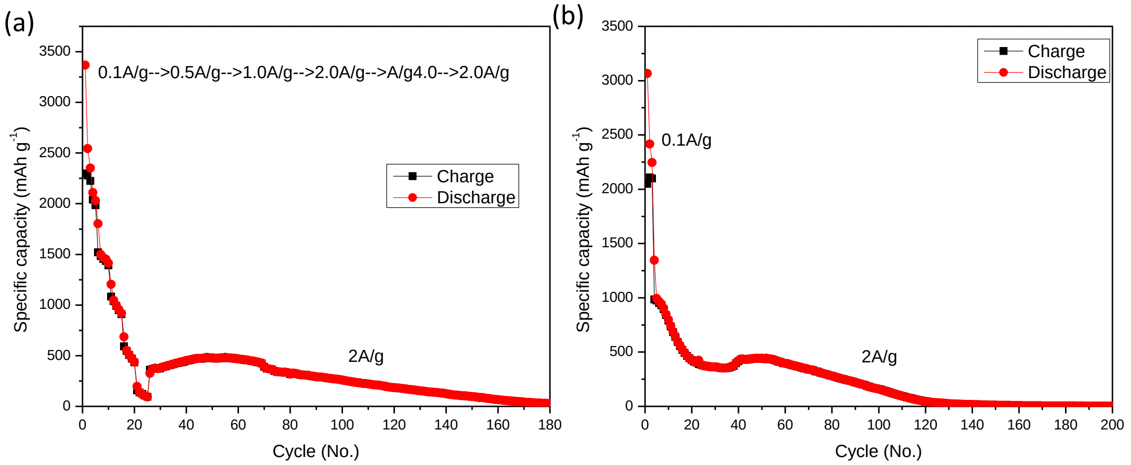

Supplementary Figure 11. Cycling performance for reference batteries. These were fabricated using SG + SiNP + PVDF with no SHT treatment. (a) The cell subjected to rate capability at different current then continued at 2 A g^{-1}. (b) The cell was tested at 0.1 A g^{-1} for 5 cycles then continued at 2 A g^{-1} for the rest.

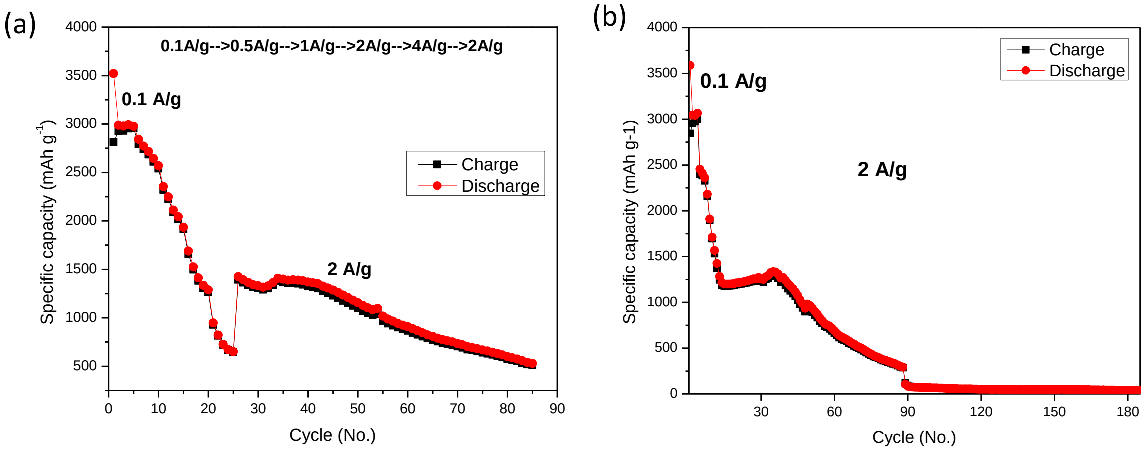

Supplementary Figure 12. **Cycling performance for reference batteries.** These were fabricated using SiNP + Graphene oxide + PAN with SHT treatment. (a) The cell subjected to rate capability at different current then continued at 2 A g^{-1}. (b) The cell was tested at 0.1 A g^{-1} for 5 cycles then continued at 2 A g^{-1} for the rest.

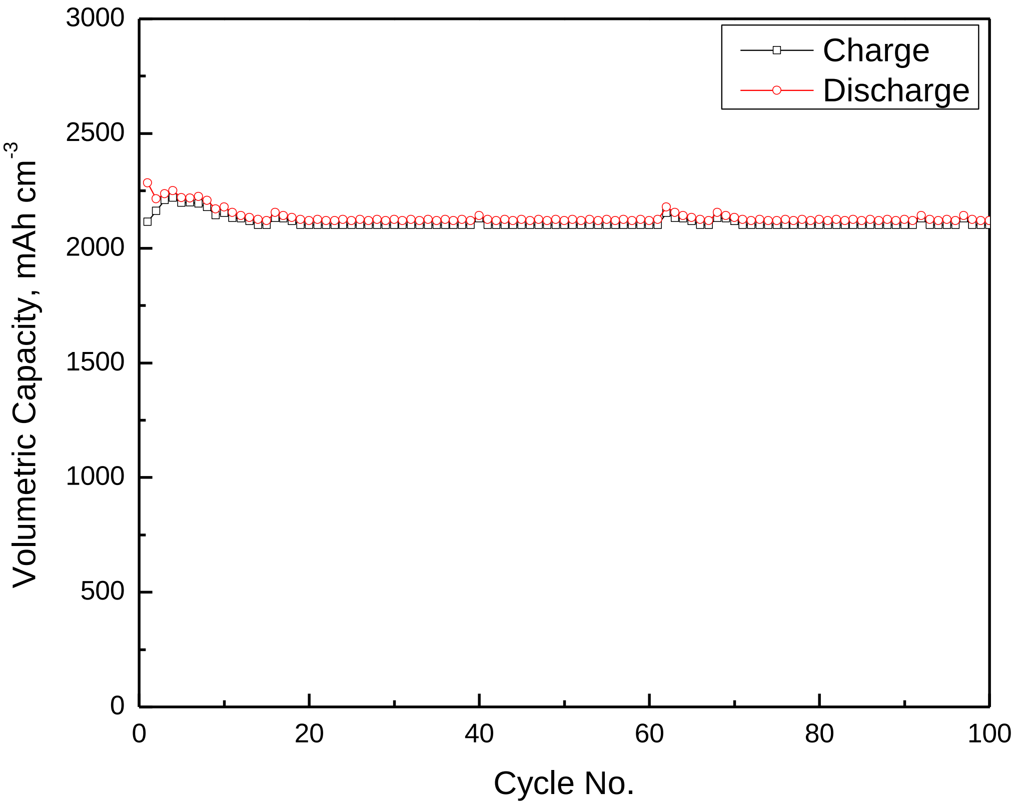

Supplementary Figure 13. **Volumetric Capacity.** The data presented here is for SG-Si-c-PAN electrode for the cell performance with data shown in Figure 4b.

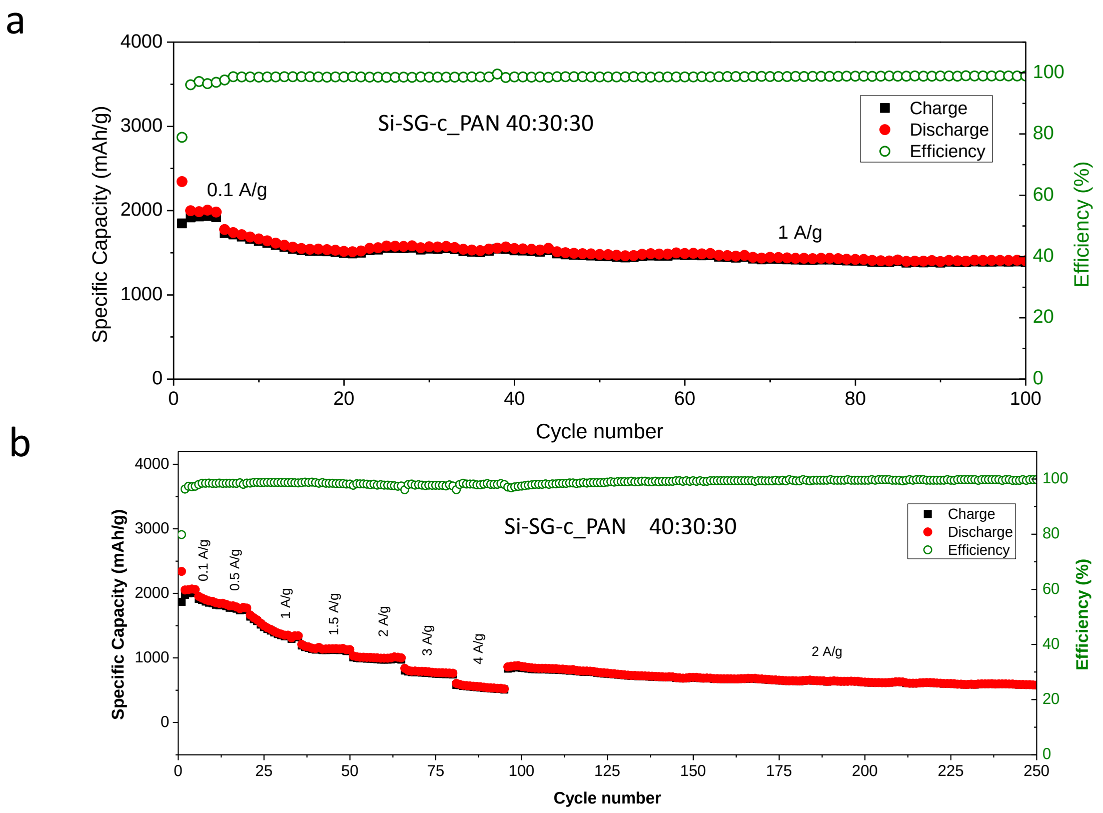

Supplementary Figure 14. **Further battery performance.** The results presented here is for SG-Si-c-PAN electrode with ratio of 40:30:30, respectively**.** (a) The cell cycled at 0.1 A g^{-1} for conditioning then continued at 1 A g^{-1}. (b) The cell started conditioning cycles then continued with rate capability at different currents then continued cycling at 2 A g^{-1}. N.B. The Capacity measured here is per mass of silicon and SG.

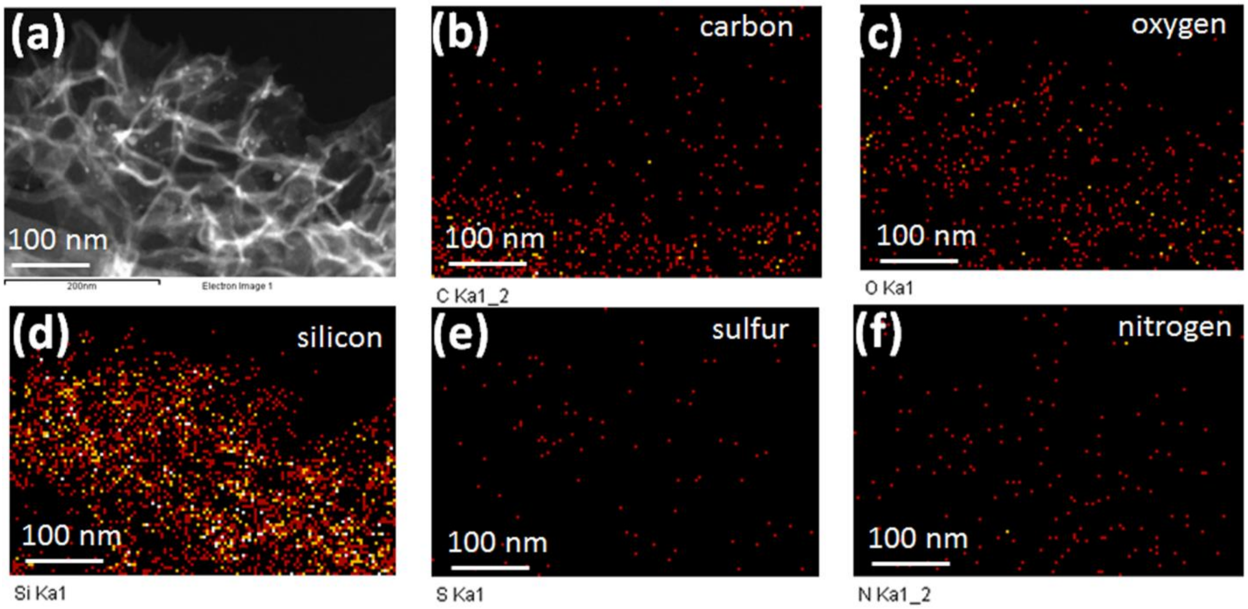

Supplementary Figure 15. **After cycling characterization of SG-Si**. (a) STEM image of SG-Si electrode material after being cycled for 2275 cycles of charge discharge, b-f) the corresponding EDX mapping of the elements carbon, oxygen, silicon, sulfur, and nitrogen, respectively.

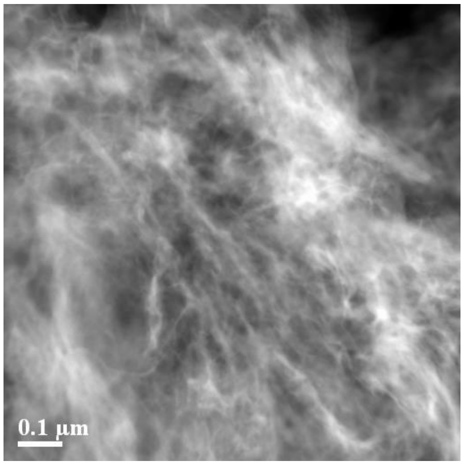

Supplementary Figure 16. After cycling characterization of G-Si. The figure shows HAADF-STEM image of the G-Si electrode after cycling for 800 cycles as shown in Figure 5e, it shows the agglomeration of Si which explain the capacity fading.

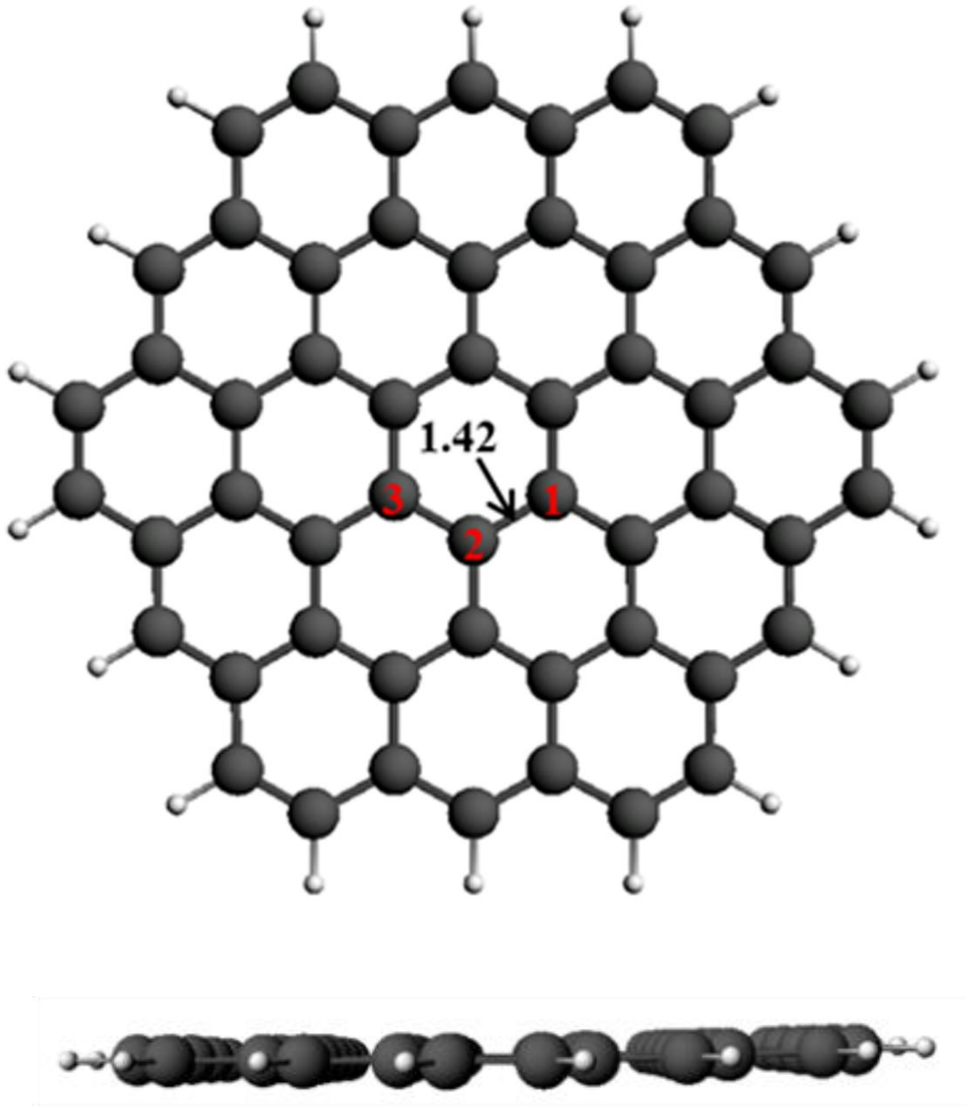

Supplementary Figure 17. **The optimized geometry of H passivated graphene (G).** Top view (top) and side view (bottom). C atoms are colored grey, H atoms white. Bond length is in angstrom.

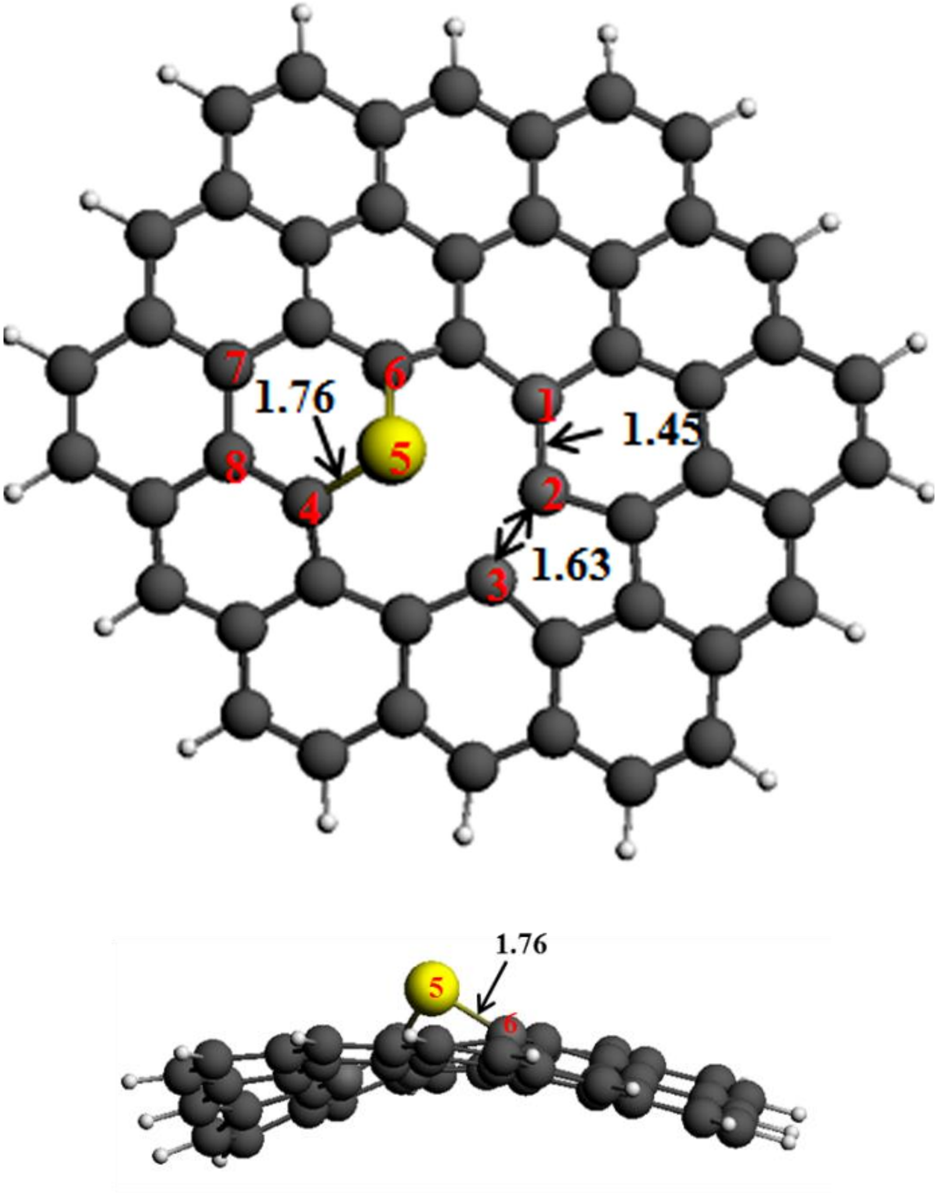

Supplementary Figure 18. **The optimized geometry of sulfur-doped graphene (S-G)**. top view (top) and side view (bottom). C atoms are colored grey, H atoms are white, S atom is yellow. Bond lengths are in angstrom.

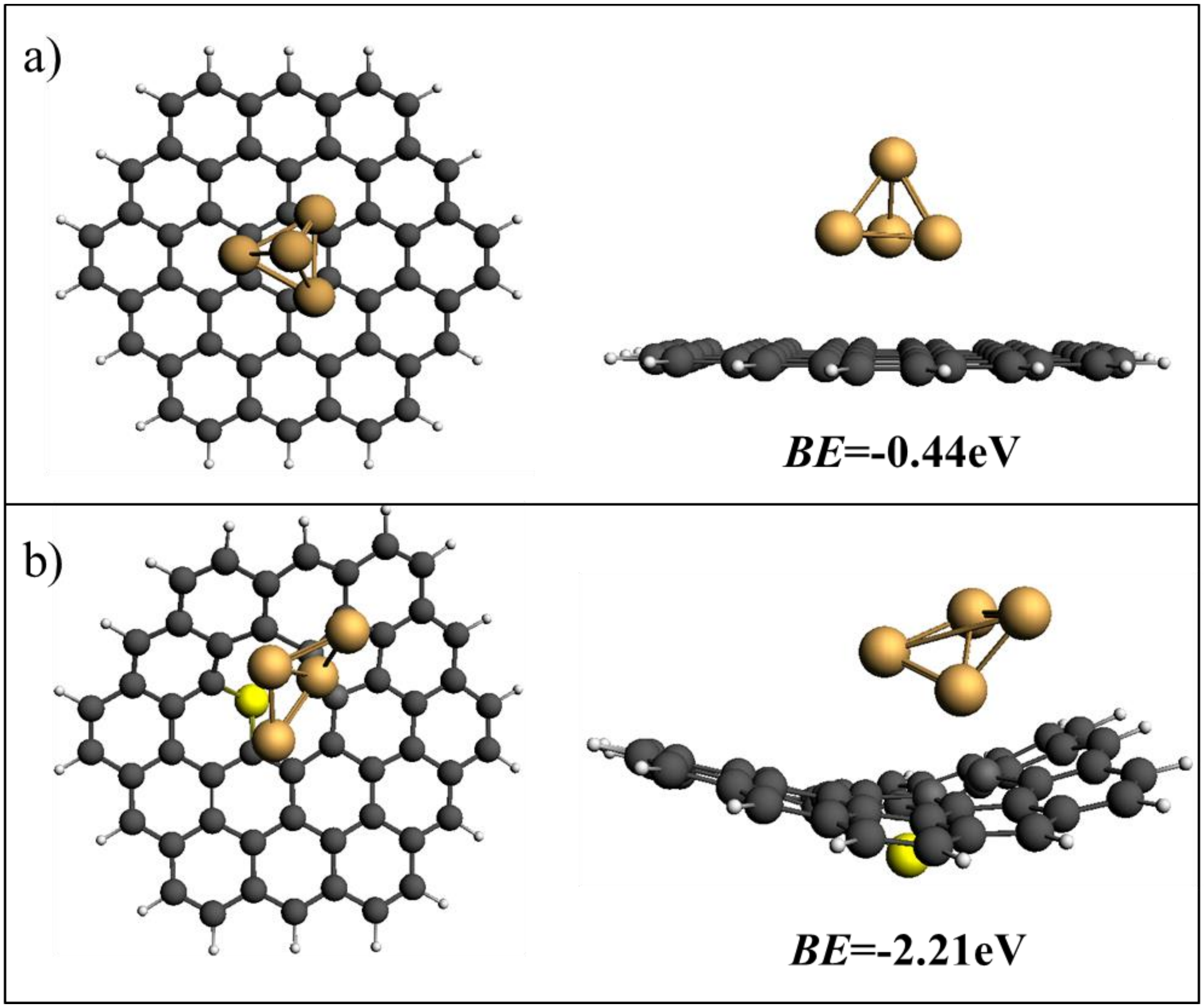

Supplementary Figure 19. **Geometries and bonding energy (*BE*) of the stable Si_4 cluster adsorption configurations.** (a) On graphene, (b) On sulfur doped graphene. C atoms are colored grey, H atoms are white, S atom is yellow, Si atoms are brown.

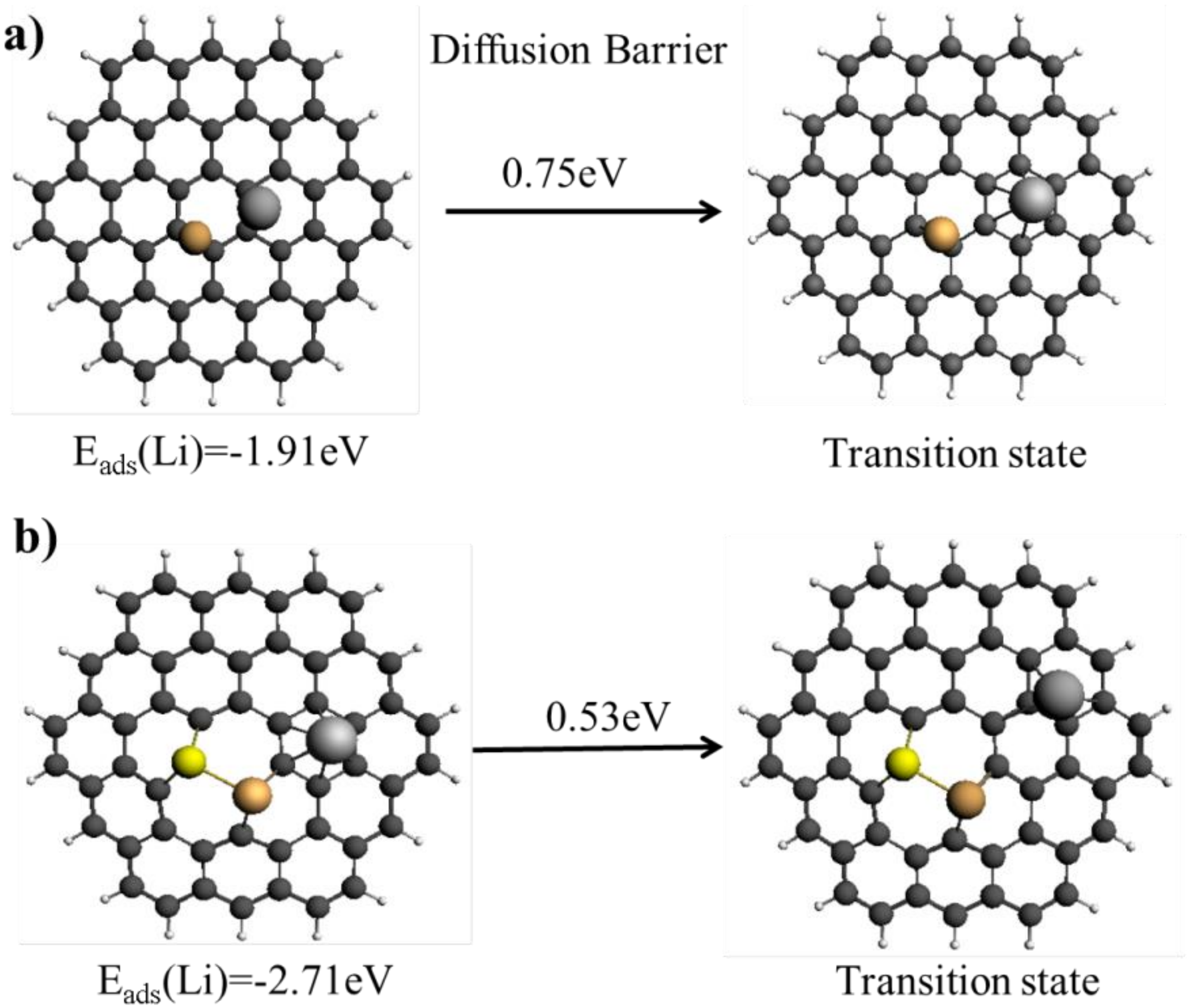

Supplementary Figure 20. **Li adsorption and transition state.** The figure quantifies Li diffusion barrier for a) G-Si and b) SG-Si
